# Census tract-level socioeconomic variables and breast cancer characteristics and outcomes in California and New York State

**DOI:** 10.1007/s10552-026-02152-1

**Published:** 2026-03-19

**Authors:** Margaret Gates Kuliszewski, Baozhen Qiao, Mandi Yu, Maria J. Schymura, Tabassum Insaf

**Affiliations:** 1 Bureau of Cancer Epidemiology, New York State Department of Health, 150 Broadway, Suite 361, Albany, NY 12204 USA; 2 Department of Epidemiology and Biostatistics, University at Albany, Albany, NY USA; 3 Division of Cancer Control and Population Sciences, National Cancer Institute, National Institutes of Health, Rockville, MD USA

**Keywords:** Breast cancer, Census tract, Health disparities, Partial synthetic data, Surveillance, Epidemiology, and End Results (SEER) program, Socioeconomic factors

## Abstract

**Purpose:**

Synthetic census tracts can allow for release of small area cancer data without compromising patient confidentiality. We used synthetic and actual census tract data for California and actual data for New York State (NYS) to examine associations of small area socioeconomic factors with breast cancer prognosis and outcomes and to evaluate results obtained from synthetic versus actual data.

**Methods:**

We retrieved data on invasive, first primary breast cancers diagnosed between 2006 and 2017 in females ages ≥ 18 in California  (*n* = 237,156) or NYS (*n* = 149,789). We categorized into quintiles census tract-level exposures and used multivariable-adjusted multilevel logistic and Cox proportional hazards regression analyses to examine associations with stage, grade, subtype, and overall and cancer-specific survival. We conducted separate analyses for California and NYS and compared results from the two states and from synthetic and actual data for California.

**Results:**

Except for income inequality, greater disadvantage for each socioeconomic variable was statistically significantly associated with more advanced stage, higher grade, higher-risk subtypes, and poorer survival in both states. Synthetic and actual results for California were consistent in direction and statistical significance, but the synthetic data tended to overestimate associations with stage and underestimate associations with grade, subtype, and survival.

**Conclusion:**

Our results indicate that residence in more disadvantaged census tracts is associated with poorer breast cancer prognosis and outcomes. Associations were similar across two large, diverse states, and synthetic results approximated actual results for California. Additional work is needed to improve early diagnosis, care, and outcomes for individuals with breast cancer in disadvantaged areas.

**Supplementary Information:**

The online version contains supplementary material available at 10.1007/s10552-026-02152-1.

## Introduction

Area-based socioeconomic measures have been associated with cancer prognosis and outcomes in multiple previous studies [1–9]. In studies of breast cancer, neighborhood-level measures including lower education and income and higher unemployment, uninsurance, and deprivation have been associated with more aggressive breast tumors [10–15], more advanced stage at diagnosis [3, 4, 16–24], and poorer survival after diagnosis [1, 2, 6, 8, 18, 19, 22, 25–33]. Fewer studies have examined measures of income inequality within a region and breast cancer outcomes, although a few studies have suggested an association between income inequality and higher breast cancer mortality in Brazil [34–36]. Although neighborhood information for cancer patients can provide valuable information, small area geographic information such as census tracts may be unavailable to researchers due to confidentiality concerns, while county-level data may not be specific enough to individual patients to provide reliable estimates of associations.

Researchers have explored possible approaches to make small area data available without jeopardizing patient confidentiality, such as the use of multiply imputed, synthetic census tract identifiers that approximate real census tracts [37]. The National Cancer Institute (NCI)’s Surveillance, Epidemiology, and End Results (SEER) program developed pilot synthetic census tract data files for California and made them available for selected projects, with NCI providing analysis validation services. In this analysis, we used California synthetic census tract data with NCI-validated analytic results and actual census tract data from New York State (NYS) to examine the association of census tract-level socioeconomic variables with breast cancer stage, grade, subtype, and survival.

California and NYS both have large, diverse populations including a high proportion of individuals born outside the U.S., with proportions of approximately 27% in California and 23% in NYS in 2023 [38, 39]. In addition, both states have a high number of new female breast cancers diagnosed each year, with 32,860 cases expected in California and 19,170 cases expected in NYS in 2025 [40]. To our knowledge, no previous studies have examined statewide associations of census tract-level socioeconomic variables with breast cancer stage, grade, subtype, and survival in California or NYS. Synthetic census tract data enable analyses of small area geographic information and cross-jurisdictional comparisons while preserving patient confidentiality, while validation analyses can assess whether synthetic data support valid statistical inference. We therefore conducted separate analyses of these associations for California and NYS and compared the results from actual census tract data for the two states and from synthetic and actual census tract data for California.

## Methods

### Study population

We retrieved data on invasive, first primary breast cancers diagnosed during 2006–2017 in females ages 18 and older, based on the years of availability of synthetic census tract data for California (SEER*Stat specialized database: California Female Malignant Breast Specialized Data with Synthetic Tract, Nov 2019 Sub, 2006–2017). We excluded cases identified by autopsy or death certificate only (*n* = 984 in California; *n* = 697 in NYS). We additionally excluded 28 NYS cases with a missing address at diagnosis because of the unavailability of census tract data and one NYS case with missing age at diagnosis. The California data did not contain any cases with missing data on census tract or age at diagnosis.

### Census tract data

Census tracts were assigned based on address at diagnosis using 2010 census geography. The methods used to create the synthetic census tract data have been described elsewhere [37]. The synthetic data for California included 10 synthetic census tracts at diagnosis for each case. Each census tract was analyzed as if it were the real census tract, and the resulting 10 sets of estimates were pooled to account for uncertainty from the synthesis process [41]. Analyses of the NYS data and California synthetic census tract data were conducted at the NYS Cancer Registry (NYSCR), while corresponding analyses of the actual census tract data for California were conducted by an honest broker under contract with NCI, Information Management Services, Inc. (IMS), using SAS code provided by the NYSCR. IMS then provided NYSCR with aggregate results for the actual California data, to allow for comparisons of the NYS and California actual results and the synthetic and actual California results.

### Exposures

The exposures of interest were socioeconomic factors assessed separately at the census tract-level, including percent living below the federal poverty line, median household income, percent unemployed, percent uninsured, percent without a high school diploma, and Gini index of income inequality, a summary measure of income dispersion across a region’s population where values range from 0 to 1 and higher values indicate greater inequality [42]. Census tract at the time of diagnosis was used to merge case data with 5-year estimates for 2009–2013 from the American Community Survey on the exposures of interest. We categorized each socioeconomic variable into quintiles, with higher quintiles corresponding to greater disadvantage or income inequality. We calculated separate quintile cut points for each state using the distribution of each variable across all census tracts in the state.

### Outcomes

Categorical outcomes included stage at diagnosis, tumor grade, and breast cancer subtype based on estrogen receptor, progesterone receptor, and HER2 status. Cases diagnosed before 2010 and cases with missing HER2 data were excluded from analyses of tumor subtype. We additionally examined associations with overall survival (OS) and cancer-specific survival (CSS) for cases diagnosed in 2006–2016 in California and 2006–2017 in NYS using a variable for number of months between date of breast cancer diagnosis and date of last known active follow up or date of death, with follow-up through December 2017 for California and December 2018 for NYS. Cases with zero days of survival time (*n* = 625 in California; *n* = 557 in NYS) and cases who were alive but had zero months of survival time (*n* = 365 in California; *n* = 503 in NYS) were omitted from the survival analyses.

### Statistical analysis

We conducted separate analyses for California and NYS for each socioeconomic variable in relation to breast cancer stage, grade, subtype, and survival using SAS version 9.4. We conducted descriptive analyses to examine the frequencies of individual-level and census tract-level variables of interest for each state. We used the SAS GLIMMIX procedure to fit multivariable-adjusted multilevel multinomial logistic regression models to examine associations between the exposures of interest and breast cancer stage, grade, and subtype, with a random intercept for census tract. For survival analyses, we used the SAS PHREG procedure to fit marginal Cox proportional hazards regression models with census tract clustering to examine associations between the census tract-level exposures of interest and OS and CSS.

We selected covariates based on a priori knowledge and omitted variables where adjustment resulted in minimal change in the associations of interest. We adjusted all analyses for age (18–40, 41–50, 51–60, 61–70, 71–80, ≥ 81 years of age), race (White, Black, Asian/Pacific Islander, Other), ethnicity (non-Hispanic, Hispanic, missing), and year of diagnosis, and additionally adjusted analyses of stage at diagnosis and survival for marital status (single, married, divorced/separated, widowed, other/unknown) and primary payer at diagnosis (uninsured, any Medicaid, all other insurance, unknown). Cases diagnosed in California in 2006 did not have primary payer information and were categorized separately. In survival analyses, we additionally adjusted for tumor stage (local, regional, distant, unknown), grade (well differentiated, moderately well differentiated, poorly/undifferentiated, unknown), and subtype [hormone receptor (HR) and HER2 positive, HR positive/HER2 negative, HR negative/HER2 positive, HR and HER2 negative, unknown], as well as separate variables for receipt of treatment (surgery, chemotherapy, radiotherapy, immunotherapy, hormonal therapy) during initial treatment, categorized as yes or no/unknown. HER2 data were unavailable for NYS cases diagnosed before 2010, and both California and NYS cases diagnosed before 2010 were categorized in a separate tumor subtype category for consistency. Additionally, information on receipt of immunotherapy and hormonal therapy was unavailable for California and these variables were omitted from survival analyses for California.

To assess possible effect modification, we additionally stratified by race and ethnicity for all NYS analyses and by breast cancer subtype for NYS survival analyses, with *p*-values for interaction calculated from likelihood ratio tests comparing models with and without interaction terms between the exposure and modifier of interest. The stratified analyses were not part of the original analysis plan and were conducted supplementally when access to the California actual census tract data was no longer available and estimates could not be verified. We therefore limited the effect modification analyses to NYS only.

Analyses conducted at the NYSCR used actual data for NYS and synthetic data for California with 10 iterations per case using simulated census tracts. We pooled results from the 10 iterations per case using the SAS MIANALYZE procedure. After completing analyses of the synthetic California data, we provided our final SAS code to IMS to allow them to run corresponding analyses of the actual California data. IMS provided aggregate results for the California actual census tract data, which were then compared with both the NYS results and supplementally with the California synthetic pooled results. All analyses used a significance level of 0.05.

## Results

Table 1 displays characteristics of the included breast cancer cases from each state. The mean age at diagnosis was similar but the race and ethnicity distribution differed, with a lower proportion of Black cases in California (6.5% vs. 15.5%) and a higher proportion of Asian/Pacific Islander (13.9% vs. 5.7%) and Hispanic (20.0% vs. 10.0%) cases, due to differences in the race and ethnicity distributions in the two states [39]. The distribution of marital status was generally similar between the two states, as was the distribution of primary payer at diagnosis after omitting California cases diagnosed in 2006, who had missing information. Comparing clinical characteristics, the distribution of stage at diagnosis was similar for the two states but with a slightly higher proportion with regional stage cancers (30.8% vs. 28.8%) and a slightly lower proportion with local, distant, and unknown stage cancers in California. California had a higher proportion of well differentiated cancers (21.6% vs. 15.5%) and a lower proportion of poorly differentiated or undifferentiated cancers (30.8% vs. 33.4%). Distribution of tumor subtype and treatment received was similar overall for the two states, although cases in California were less likely to have received radiation therapy during initial therapy (46.5% vs. 55.2%). A higher proportion of NYS cases were deceased at the time of analysis, due at least in part to the inclusion of an additional year of follow-up for NYS cases. Although we conducted separate analyses for California and NYS, in a combined analysis there were statistically significant differences between the two states in the distributions of all variables in Table 1 except for receipt of chemotherapy during initial treatment (chi-square *p* = 0.25).

**Table 1:** Characteristics of invasive breast cancer cases diagnosed in 2006–2017 in California and New York State

Characteristic	California	New York
Number of cases	237,156	149,789
Mean (SD) age in years	60.4 (13.8)	60.8 (14.0)
Race, *n* (%)		
White	184,859 (78.0)	116,521 (77.8)
Black	15,400 (6.5)	23,190 (15.5)
Asian/Pacific Islander	33,054 (13.9)	8,583 (5.7)
Other/unknown^a^	3,843 (1.6)	1,495 (1.0)
Hispanic ethnicity, *n* (%)	47,480 (20.0)	14,980 (10.0)
Marital status, *n* (%)		
Single	38,444 (16.2)	29,235 (19.5)
Married	129,970 (54.8)	76,418 (51.0)
Divorced or separated	27,297 (11.5)	17,206 (11.5)
Widowed	29,779 (12.6)	21,889 (14.6)
Other or unknown	11,666 (4.9)	5,041 (3.4)
Primary payer at diagnosis, *n* (%)^b^		
Uninsured	2,431 (1.0)	1,860 (1.2)
Any Medicaid	31,909 (13.5)	22,578 (15.1)
Insured excluding Medicaid	177,335 (74.8)	122,362 (81.7)
Unknown	7,648 (3.2)	2,989 (2.0)
Cases diagnosed in 2006	17,833 (7.5)	–
Disease stage at diagnosis, *n* (%)		
Local	147,497 (62.2)	94,060 (62.8)
Regional	72,970 (30.8)	43,179 (28.8)
Distant	12,590 (5.3)	8,717 (5.8)
Unknown	4,099 (1.7)	3,833 (2.6)
Tumor differentiation, *n* (%)		
Well differentiated	51,185 (21.6)	23,245 (15.5)
Moderately well differentiated	97,105 (41.0)	64,019 (42.7)
Poorly/undifferentiated	73,116 (30.8)	50,047 (33.4)
Unknown	15,750 (6.6)	12,478 (8.3)
Tumor subtype, *n* (%)^c^		
HR+/HER2+ (Luminal B)	16,835 (7.1)	9,862 (6.6)
HR+/HER2− (Luminal A)	109,231 (46.1)	66,102 (44.1)
HR−/HER2+ (HER2-enriched)	7,240 (3.1)	4,372 (2.9)
HR−/HER2− (triple negative)	15,919 (6.7)	10,356 (6.9)
Unknown	13,771 (5.8)	10,878 (7.3)
Cases diagnosed prior to 2010	74,160 (31.3)	48,219 (32.2)
Received surgery during initial treatment, *n* (%)	214,625 (90.5)	134,235 (89.6)
Received chemotherapy during initial treatment, *n* (%)	94,762 (40.0)	60,132 (40.1)
Received radiation during initial treatment, *n* (%)	110,173 (46.5)	82,612 (55.2)
Received hormonal therapy during initial treatment, *n* (%)	NA	78,026 (52.1)
Received immunotherapy during initial treatment, *n* (%)	NA	7,580 (5.1)
Deceased, *n* (%)^d^	41,014 (17.3)	30,880 (20.6)

Percentages may not sum to 100 due to rounding. *NA* not available

^a^Includes individuals with reported race of “American Indian or Alaska Native”, “some other race”, or “unknown by patient”. These categories were combined because of a small number of cases

^b^Primary payer is not available for California cases diagnosed in 2006

^c^Hormone receptor (HR) positive = estrogen receptor and/or progesterone receptor positive. HER2 status is missing for all New York State (NYS) cases diagnosed prior to 2010

^d^California patients were followed through December 2017; NYS patients were followed through December 2018

In multivariable-adjusted logistic regression analyses, residence in a census tract with higher percent living below the poverty line, percent unemployed, percent uninsured, percent without a high school diploma, or lower median income was associated with more advanced stage at diagnosis in both states (Table 2). In California, the strongest associations were observed for percent without a high school diploma, with odds ratios (OR) and 95% confidence intervals (95% CI) for the highest versus lowest quintile of 1.20 (95% CI 1.16–1.24) for regional and 1.47 (95% CI 1.38–1.57) for distant versus local stage cancers. Associations were similar in magnitude and direction for percent uninsured, percent below the poverty line, and median household income, and were slightly lower but still statistically significant for percent uninsured. In NYS, percent without a high school diploma was again most strongly associated with advanced stage at diagnosis (for highest vs. lowest quintile, OR = 1.24, 95% CI 1.19–1.30 for regional and OR = 1.49, 95% CI 1.37–1.62 for distant vs. local stage cancers). Higher percent uninsured, percent unemployed, percent below the poverty line, and lower median household income were all associated with statistically significant higher odds of regional and distant stage cancer. Income inequality was not clearly associated with more advanced stage at diagnosis in either state, although there was a statistically significant association between highest versus lowest quintile of inequality and lower odds of distant versus local stage cancer in NYS (OR = 0.90, 95% CI 0.84–0.97).

**Table 2:** Multivariable-adjusted odds ratios (OR) and 95% confidence intervals (CI) for associations of census tract-level socioeconomic variables and breast cancer stage at diagnosis for invasive breast cancer cases diagnosed in 2006–2017 in California and New York State

Census tract-level exposure variables	California			New York		
	Stage at diagnosis^a^			Stage at diagnosis^a^		
	Regional	Distant	Unknown	Regional	Distant	Unknown
Percent below poverty line						
Q1 (lowest %)	1.00 (ref)	1.00 (ref)	1.00 (ref)	1.00 (ref)	1.00 (ref)	1.00 (ref)
Q2	1.02 (0.99–1.05)	**1.09 (1.02–1.17)**	**1.18 (1.04–1.33)**	**1.05 (1.01–1.10)**	1.05 (0.97–1.13)	1.01 (0.89–1.14)
Q3	**1.07 (1.04–1.10)**	**1.17 (1.10–1.25)**	**1.32 (1.17–1.49)**	**1.09 (1.05–1.14)**	**1.15 (1.07–1.24)**	0.98 (0.87–1.11)
Q4	**1.12 (1.09–1.16)**	**1.25 (1.17–1.33)**	**1.41 (1.25–1.59)**	**1.14 (1.09, 1.18)**	**1.21 (1.12, 1.31)**	0.99 (0.87–1.12)
Q5 (highest %)	**1.16 (1.13–1.20)**	**1.39 (1.31–1.48)**	**1.70 (1.51–1.91)**	**1.19 (1.14–1.24)**	**1.27 (1.17–1.37)**	0.98 (0.86–1.11)
Median household income						
Q1 (highest income)	1.00 (ref)	1.00 (ref)	1.00 (ref)	1.00 (ref)	1.00 (ref)	1.00 (ref)
Q2	1.03 (1.00–1.06)	**1.14 (1.06–1.21)**	1.10 (0.97–1.25)	**1.08 (1.04–1.12)**	**1.14 (1.06–1.23)**	1.10 (0.97–1.24)
Q3	**1.08 (1.05–1.11)**	**1.15 (1.08–1.23)**	**1.18 (1.05–1.34)**	**1.15 (1.11–1.20)**	**1.15 (1.06–1.24)**	1.04 (0.91–1.17)
Q4	**1.14 (1.11–1.18)**	**1.33 (1.24–1.41)**	**1.42 (1.26–1.60)**	**1.13 (1.08–1.17)**	**1.16 (1.08–1.26)**	1.03 (0.91–1.17)
Q5 (lowest income)	**1.17 (1.13–1.20)**	**1.41 (1.32–1.50)**	**1.65 (1.47–1.86)**	**1.20 (1.15–1.25)**	**1.28 (1.18–1.38)**	1.05 (0.92–1.19)
Gini index of income inequality						
Q1 (lowest inequality)	1.00 (ref)	1.00 (ref)	1.00 (ref)	1.00 (ref)	1.00 (ref)	1.00 (ref)
Q2	1.02 (0.99–1.05)	1.05 (0.99–1.12)	1.11 (0.99–1.24)	1.01 (0.98–1.05)	0.96 (0.89–1.03)	0.99 (0.87–1.12)
Q3	1.02 (0.99–1.05)	1.05 (0.98–1.11)	1.08 (0.96–1.21)	1.00 (0.96–1.04)	**0.91 (0.85–0.98)**	0.97 (0.86–1.10)
Q4	1.00 (0.97–1.03)	1.05 (0.99–1.11)	1.08 (0.97–1.21)	0.99 (0.95–1.03)	0.96 (0.89–1.03)	0.98 (0.87–1.11)
Q5 (highest inequality)	0.97 (0.94–1.00)	0.95 (0.89–1.01)	**1.13 (1.01–1.27)**	0.97 (0.93–1.00)	**0.90 (0.84–0.97)**	0.92 (0.81–1.04)
Percent unemployed						
Q1 (lowest %)	1.00 (ref)	1.00 (ref)	1.00 (ref)	1.00 (ref)	1.00 (ref)	1.00 (ref)
Q2	1.02 (0.99–1.05)	**1.09 (1.02–1.16)**	**1.17 (1.03–1.32)**	1.03 (0.99–1.07)	**1.16 (1.08–1.25)**	1.08 (0.95–1.22)
Q3	**1.08 (1.05–1.11)**	**1.13 (1.06–1.20)**	**1.16 (1.02–1.31)**	**1.05 (1.01–1.09)**	**1.19 (1.11–1.29)**	1.09 (0.96–1.24)
Q4	**1.12 (1.09–1.16)**	**1.21 (1.13–1.28)**	**1.33 (1.18–1.50)**	**1.12 (1.07–1.16)**	**1.22 (1.13–1.31)**	**1.15 (1.02–1.30)**
Q5 (highest %)	**1.14 (1.11–1.17)**	**1.28 (1.20–1.36)**	**1.52 (1.35–1.70)**	**1.14 (1.10–1.19)**	**1.29 (1.20–1.40)**	1.06 (0.93–1.21)
Percent uninsured						
Q1 (lowest %)	1.00 (ref)	1.00 (ref)	1.00 (ref)	1.00 (ref)	1.00 (ref)	1.00 (ref)
Q2	1.03 (1.00–1.06)	**1.12 (1.05–1.20)**	**1.33 (1.17–1.51)**	**1.05 (1.01–1.09)**	**1.18 (1.09–1.27)**	1.04 (0.92–1.18)
Q3	**1.08 (1.05–1.11)**	**1.23 (1.16–1.32)**	**1.34 (1.19–1.52)**	**1.11 (1.07–1.16)**	**1.23 (1.14–1.33)**	0.97 (0.86–1.10)
Q4	**1.13 (1.10–1.17)**	**1.37 (1.29–1.46)**	**1.40 (1.24–1.58)**	**1.18 (1.14–1.23)**	**1.34 (1.24–1.45)**	0.96 (0.85–1.09)
Q5 (highest %)	**1.19 (1.15–1.22)**	**1.39 (1.31–1.49)**	**1.82 (1.61–2.05)**	**1.20 (1.15–1.25)**	**1.39 (1.28–1.50)**	1.04 (0.92–1.19)
Percent without high school diploma						
Q1 (lowest %)	1.00 (ref)	1.00 (ref)	1.00 (ref)	1.00 (ref)	1.00 (ref)	1.00 (ref)
Q2	**1.06 (1.02–1.09)**	**1.14 (1.07–1.22)**	**1.21 (1.07–1.37)**	**1.10 (1.05–1.14)**	**1.20 (1.11–1.30)**	**1.15 (1.01–1.31)**
Q3	**1.11 (1.07–1.14)**	**1.27 (1.20–1.36)**	**1.22 (1.08–1.38)**	**1.13 (1.09–1.18)**	**1.31 (1.21–1.41)**	**1.19 (1.04–1.35)**
Q4	**1.14 (1.11–1.18)**	**1.32 (1.24–1.40)**	**1.41 (1.25–1.59)**	**1.20 (1.15–1.25)**	**1.46 (1.35–1.58)**	**1.29 (1.13–1.46)**
Q5 (highest %)	**1.20 (1.16–1.24)**	**1.47 (1.38–1.57)**	**1.65 (1.46–1.86)**	**1.24 (1.19–1.30)**	**1.49 (1.37–1.62)**	**1.26 (1.10–1.44)**

Analyses adjusted for age, race, ethnicity, marital status, primary payer, and year of diagnosis. Statistically significant associations are shown in bold

^a^Reference category for stage at diagnosis is local stage disease

Comparing well or moderately well differentiated tumors with poorly differentiated/undifferentiated tumors and tumors with unknown differentiation, we observed statistically significant associations with nearly all socioeconomic variables (Table 3), indicating that living in a more disadvantaged census tract is associated with higher odds of being diagnosed with less differentiated tumors. In general, associations were stronger in California versus NYS, except for income inequality where there was a statistically significant association in NYS but not California. In both states the strongest associations were observed for higher percent uninsured, percent without a high school diploma, percent below the poverty line, and lower median household income.

**Table 3:** Multivariable-adjusted odds ratios (OR) and 95% confidence intervals (CI) for associations of census tract-level socioeconomic variables and breast tumor differentiation for invasive breast cancer cases diagnosed in 2006–2017 in California and New York State

Census tract-level exposure variables	California		New York	
	Tumor differentiation^a^		Tumor differentiation^a^	
	Poorly differentiated/undifferentiated	Unknown	Poorly differentiated/undifferentiated	Unknown
Percent below poverty line				
Q1 (lowest %)	1.00 (ref)	1.00 (ref)	1.00 (ref)	1.00 (ref)
Q2	**1.04 (1.01–1.07)**	1.05 (0.99–1.12)	1.01 (0.97–1.05)	1.03 (0.95–1.10)
Q3	**1.09 (1.06–1.13)**	**1.17 (1.10–1.25)**	**1.06 (1.02–1.10)**	1.06 (0.98–1.13)
Q4	**1.19 (1.15–1.23)**	**1.34 (1.26–1.42)**	**1.09 (1.05–1.13)**	**1.15 (1.07–1.23)**
Q5 (highest %)	**1.29 (1.25–1.33)**	**1.50 (1.41–1.59)**	**1.14 (1.09–1.19)**	**1.28 (1.19–1.38)**
Median household income				
Q1 (highest income)	1.00 (ref)	1.00 (ref)	1.00 (ref)	1.00 (ref)
Q2	**1.09 (1.06–1.13)**	**1.12 (1.05–1.19)**	1.03 (0.99–1.07)	0.93 (0.86–1.00)
Q3	**1.16 (1.12–1.20)**	**1.21 (1.14–1.29)**	**1.05 (1.01–1.09)**	**0.89 (0.83–0.95)**
Q4	**1.26 (1.22–1.30)**	**1.41 (1.33–1.50)**	**1.11 (1.07–1.15)**	**0.92 (0.85–0.99)**
Q5 (lowest income)	**1.36 (1.31–1.40)**	**1.55 (1.46–1.65)**	**1.12 (1.07–1.16)**	1.07 (1.00–1.15)
Gini index of income inequality				
Q1 (lowest inequality)	1.00 (ref)	1.00 (ref)	1.00 (ref)	1.00 (ref)
Q2	1.02 (0.99–1.06)	1.04 (0.98–1.10)	1.03 (0.99–1.07)	1.06 (0.99–1.14)
Q3	1.02 (0.99–1.05)	1.03 (0.97–1.09)	1.03 (0.99–1.07)	**1.13 (1.06–1.22)**
Q4	1.03 (0.99–1.06)	1.05 (0.99–1.12)	**1.09 (1.05–1.13)**	**1.28 (1.20–1.38)**
Q5 (highest inequality)	0.97 (0.94–1.00)	0.94 (0.88–1.00)	**1.08 (1.03–1.12)**	**1.34 (1.25–1.44)**
Percent unemployed				
Q1 (lowest %)	1.00 (ref)	1.00 (ref)	1.00 (ref)	1.00 (ref)
Q2	1.03 (1.00–1.07)	1.06 (1.00–1.12)	1.03 (0.99–1.07)	**1.08 (1.01–1.16)**
Q3	**1.09 (1.05–1.12)**	**1.15 (1.08–1.22)**	1.02 (0.98–1.06)	**1.11 (1.03–1.19)**
Q4	**1.15 (1.12–1.19)**	**1.27 (1.19–1.34)**	**1.08 (1.03–1.12)**	**1.20 (1.12–1.29)**
Q5 (highest %)	**1.20 (1.17–1.24)**	**1.50 (1.41–1.59)**	**1.07 (1.03–1.12)**	**1.23 (1.14–1.32)**
Percent uninsured				
Q1 (lowest %)	1.00 (ref)	1.00 (ref)	1.00 (ref)	1.00 (ref)
Q2	**1.12 (1.08–1.15)**	**1.17 (1.10–1.24)**	1.00 (0.96–1.04)	0.98 (0.91–1.05)
Q3	**1.17 (1.14–1.21)**	**1.30 (1.23–1.39)**	1.04 (1.00–1.08)	1.07 (1.00–1.15)
Q4	**1.25 (1.21–1.29)**	**1.39 (1.31–1.48)**	**1.11 (1.06–1.15)**	**1.11 (1.03–1.19)**
Q5 (highest %)	**1.39 (1.35–1.44)**	**1.49 (1.40–1.59)**	**1.13 (1.09–1.18)**	**1.25 (1.17–1.35)**
Percent without high school diploma				
Q1 (lowest %)	1.00 (ref)	1.00 (ref)	1.00 (ref)	1.00 (ref)
Q2	**1.08 (1.04–1.11)**	**1.18 (1.11–1.25)**	1.03 (0.99–1.07)	1.02 (0.95–1.10)
Q3	**1.15 (1.11–1.19)**	**1.31 (1.23–1.39)**	**1.05 (1.01–1.09)**	1.03 (0.96–1.11)
Q4	**1.24 (1.20–1.28)**	**1.42 (1.34–1.51)**	**1.13 (1.09–1.18)**	**1.17 (1.09–1.26)**
Q5 (highest %)	**1.37 (1.32–1.41)**	**1.56 (1.46–1.66)**	**1.14 (1.09–1.19)**	**1.35 (1.25–1.46)**

Analyses adjusted for age, race, ethnicity, and year of diagnosis. Statistically significant associations are shown in bold

^a^Reference category for tumor differentiation is well/moderately well differentiated

Similarly, for tumor subtype we observed statistically significant associations between all socioeconomic variables except for income inequality (Table 4). Living in a more disadvantaged census tract was associated with higher odds of being diagnosed with HR+/HER2+ (Luminal B), HR−/HER2+ (HER2-enriched), and HR−/HER2− (triple negative) versus HR+/HER2− (Luminal A) tumors. The associations were similar across the two states, although in NYS the associations tended to be strongest for triple negative tumors, while for California the associations were generally strongest for HER2-enriched tumors.

**Table 4:** Multivariable-adjusted odds ratios (OR) and 95% confidence intervals (CI) for associations of census tract-level socioeconomic variables and breast tumor subtype for invasive breast cancer cases diagnosed in 2010–2017 in California and New York State

Census tract-level exposure variables	California			New York		
	Tumor subtype^a^			Tumor subtype^a^		
	HR+/HER2+ (Luminal B)	HR−/HER2+ (HER2-enriched)	HR−/HER2- (triple negative)	HR+/HER2+ (Luminal B)	HR−/HER2+ (HER2-enriched)	HR−/HER2− (triple negative)
Percent below poverty line						
Q1 (lowest %)	1.00 (ref)	1.00 (ref)	1.00 (ref)	1.00 (ref)	1.00 (ref)	1.00 (ref)
Q2	0.99 (0.94–1.05)	1.06 (0.98–1.15)	1.05 (0.99–1.11)	1.02 (0.95–1.09)	1.02 (0.92–1.13)	1.05 (0.98–1.13)
Q3	1.05 (0.99–1.10)	**1.12 (1.03–1.21)**	**1.13 (1.07–1.19)**	1.06 (0.99–1.13)	**1.13 (1.02–1.25)**	**1.11 (1.03–1.19)**
Q4	**1.07 (1.01–1.13)**	**1.20 (1.11–1.30)**	**1.16 (1.10–1.23)**	**1.14 (1.06–1.22)**	**1.12 (1.01–1.24)**	**1.20 (1.12–1.28)**
Q5 (highest %)	**1.13 (1.07–1.19)**	**1.33 (1.23–1.44)**	**1.28 (1.21–1.36)**	**1.13 (1.05–1.21)**	**1.17 (1.05–1.30)**	**1.31 (1.22–1.41)**
Median household income						
Q1 (highest income)	1.00 (ref)	1.00 (ref)	1.00 (ref)	1.00 (ref)	1.00 (ref)	1.00 (ref)
Q2	1.04 (0.99–1.10)	**1.09 (1.01–1.18)**	**1.07 (1.01–1.13)**	1.04 (0.97–1.11)	**1.13 (1.02–1.25)**	**1.15 (1.07–1.24)**
Q3	**1.08 (1.02–1.14)**	**1.18 (1.09–1.27)**	**1.12 (1.06–1.19)**	**1.09 (1.01–1.17)**	**1.21 (1.09–1.33)**	**1.21 (1.13–1.30)**
Q4	**1.11 (1.05–1.17)**	**1.22 (1.13–1.32)**	**1.23 (1.16–1.30)**	**1.09 (1.01–1.17)**	**1.17 (1.06–1.30)**	**1.30 (1.21–1.39)**
Q5 (lowest income)	**1.17 (1.11–1.24)**	**1.36 (1.25–1.47)**	**1.29 (1.22–1.36)**	**1.11 (1.03–1.19)**	**1.25 (1.12–1.38)**	**1.41 (1.31–1.51)**
Gini index of income inequality						
Q1 (lowest inequality)	1.00 (ref)	1.00 (ref)	1.00 (ref)	1.00 (ref)	1.00 (ref)	1.00 (ref)
Q2	1.03 (0.98–1.09)	1.05 (0.97–1.13)	1.01 (0.96–1.07)	**1.12 (1.04–1.20)**	0.99 (0.90–1.09)	1.03 (0.97–1.11)
Q3	0.97 (0.92–1.02)	1.07 (1.00–1.15)	0.98 (0.93–1.03)	1.05 (0.98–1.13)	**0.88 (0.80–0.98)**	0.98 (0.92–1.05)
Q4	1.00 (0.95–1.05)	0.99 (0.92–1.07)	0.98 (0.93–1.04)	**1.11 (1.03–1.19)**	0.96 (0.87–1.06)	1.06 (0.99–1.13)
Q5 (highest inequality)	1.01 (0.96–1.06)	0.95 (0.88–1.02)	**0.90 (0.86–0.95)**	1.02 (0.95–1.10)	0.93 (0.84–1.02)	0.97 (0.91–1.04)
Percent unemployed						
Q1 (lowest %)	1.00 (ref)	1.00 (ref)	1.00 (ref)	1.00 (ref)	1.00 (ref)	1.00 (ref)
Q2	1.00 (0.95–1.05)	1.06 (0.98–1.14)	**1.12 (1.06–1.18)**	1.04 (0.97–1.11)	**1.16 (1.05–1.29)**	1.03 (0.96–1.11)
Q3	**1.08 (1.02–1.14)**	**1.09 (1.01–1.18)**	**1.13 (1.07–1.19)**	1.03 (0.96–1.11)	**1.13 (1.02–1.25)**	**1.09 (1.01–1.17)**
Q4	**1.11 (1.05–1.17)**	**1.16 (1.07–1.25)**	**1.19 (1.13–1.26)**	1.05 (0.98–1.13)	**1.20 (1.08–1.33)**	**1.11 (1.04–1.19)**
Q5 (highest %)	**1.06 (1.01–1.12)**	**1.26 (1.16–1.36)**	**1.26 (1.19–1.33)**	**1.10 (1.02–1.18)**	**1.15 (1.04–1.28)**	**1.22 (1.14–1.31)**
Percent uninsured						
Q1 (lowest %)	1.00 (ref)	1.00 (ref)	1.00 (ref)	1.00 (ref)	1.00 (ref)	1.00 (ref)
Q2	1.02 (0.96–1.07)	**1.12 (1.03–1.21)**	**1.13 (1.07–1.19)**	0.99 (0.92–1.06)	1.04 (0.94–1.16)	1.04 (0.97–1.12)
Q3	**1.10 (1.04–1.16)**	**1.19 (1.10–1.29)**	**1.18 (1.12–1.25)**	1.02 (0.95–1.10)	1.09 (0.98–1.20)	**1.13 (1.05–1.21)**
Q4	**1.10 (1.04–1.16)**	**1.29 (1.19–1.39)**	**1.22 (1.15–1.29)**	1.07 (1.00–1.15)	**1.18 (1.07–1.31)**	**1.22 (1.13–1.31)**
Q5 (highest %)	**1.18 (1.12–1.25)**	**1.34 (1.23–1.45)**	**1.28 (1.21–1.36)**	**1.15 (1.06–1.23)**	**1.20 (1.08–1.33)**	**1.17 (1.09–1.26)**
Percent without high school diploma						
Q1 (lowest %)	1.00 (ref)	1.00 (ref)	1.00 (ref)	1.00 (ref)	1.00 (ref)	1.00 (ref)
Q2	1.05 (0.99–1.10)	**1.10 (1.02–1.19)**	**1.10 (1.04–1.16)**	0.99 (0.92–1.06)	1.05 (0.94–1.16)	**1.11 (1.04–1.20)**
Q3	**1.09 (1.04–1.15)**	**1.22 (1.13–1.32)**	**1.18 (1.11–1.25)**	1.03 (0.96–1.11)	**1.22 (1.10–1.35)**	**1.18 (1.10–1.27)**
Q4	**1.12 (1.06–1.19)**	**1.24 (1.15–1.35)**	**1.25 (1.18–1.32)**	**1.13 (1.05–1.21)**	**1.18 (1.06–1.30)**	**1.26 (1.17–1.35)**
Q5 (highest %)	**1.18 (1.12–1.25)**	**1.38 (1.27–1.49)**	**1.37 (1.29–1.45)**	**1.14 (1.06–1.23)**	**1.25 (1.13–1.40)**	**1.39 (1.29–1.50)**

Analyses adjusted for age, race, ethnicity, and year of diagnosis. Statistically significant associations are shown in bold

^a^Reference category for tumor subtype is HR+/HER2− (Luminal A). Cases diagnosed before 2010 were excluded and cases with missing or unknown HR or HER2 status are omitted from table

In survival analyses, we observed statistically significant associations between higher levels of disadvantage for all socioeconomic variables except income inequality and poorer survival (Table 5). In California, the strongest associations were observed for the highest versus lowest quintile of percent without a high school diploma, with hazard ratios (HR) of 1.41 (95% CI 1.35–1.47) for OS and 1.38 (95% CI 1.31–1.46) for CSS, median household income (HR_OS_ = 1.38, 95% CI 1.33–1.44; HR_CSS_ = 1.35, 95% CI 1.29–1.42), and percent uninsured (HR_OS_ = 1.36, 95% CI 1.31–1.41; HR_CSS_ = 1.37, 95% CI 1.30–1.43). Similarly, in NYS the strongest associations were observed for the highest versus lowest quintile of median household income (HR_OS_ = 1.29, 95% CI 1.23–1.35; HR_CSS_ = 1.24, 95% CI 1.17–1.31) and percent without a high school diploma (HR_OS_ = 1.27, 95% CI 1.21–1.33; HR_CSS_ = 1.18, 95% CI 1.11–1.26). For census tracts with higher income inequality there was evidence of a survival benefit, with HRs for the highest versus lowest quintile of income inequality of 0.92 (95% CI 0.89–0.96) for OS in California, 0.91 (95% CI 0.87–0.96) for CSS in California, 0.92 (95% CI 0.88–0.96) for OS in NYS, and 0.95 (95% CI 0.90–1.01) for CSS in NYS. The survival analysis results were generally similar for the two states, but associations tended to be stronger in California than in NYS.

**Table 5:** Multivariable-adjusted hazard ratios (HR) and 95% confidence intervals (CI) for associations of census tract-level socioeconomic variables and overall and cancer-specific survival for invasive breast cancer cases diagnosed in 2006–2016 in California and 2006–2017 in New York State

Census tract-level exposure variables	California		New York	
	Overall survival	Cancer-specific survival	Overall survival	Cancer-specific survival
Percent below poverty line				
Q1 (lowest %)	1.00 (ref)	1.00 (ref)	1.00 (ref)	1.00 (ref)
Q2	**1.06 (1.02–1.10)**	**1.06 (1.01–1.12)**	**1.06 (1.02–1.11)**	1.03 (0.97–1.09)
Q3	**1.11 (1.06–1.15)**	**1.10 (1.04–1.15)**	**1.11 (1.06–1.16)**	**1.07 (1.01–1.13)**
Q4	**1.21 (1.16–1.25)**	**1.20 (1.14–1.26)**	**1.15 (1.10–1.20)**	**1.13 (1.07–1.19)**
Q5 (highest %)	**1.31 (1.26–1.36)**	**1.30 (1.24–1.36)**	**1.23 (1.17–1.29)**	**1.16 (1.10–1.23)**
Median household income				
Q1 (highest income)	1.00 (ref)	1.00 (ref)	1.00 (ref)	1.00 (ref)
Q2	**1.12 (1.08–1.17)**	**1.10 (1.05–1.16)**	**1.11 (1.06–1.16)**	**1.08 (1.02–1.14)**
Q3	**1.20 (1.16–1.25)**	**1.18 (1.12–1.24)**	**1.22 (1.17–1.27)**	**1.17 (1.11–1.24)**
Q4	**1.30 (1.25–1.35)**	**1.27 (1.21–1.33)**	**1.24 (1.19–1.30)**	**1.20 (1.13–1.27)**
Q5 (lowest income)	**1.38 (1.33–1.44)**	**1.35 (1.29–1.42)**	**1.29 (1.23–1.35)**	**1.24 (1.17–1.31)**
Gini index of income inequality				
Q1 (lowest inequality)	1.00 (ref)	1.00 (ref)	1.00 (ref)	1.00 (ref)
Q2	1.01 (0.98–1.05)	0.98 (0.93–1.02)	0.99 (0.94–1.03)	0.98 (0.93–1.03)
Q3	1.01 (0.98–1.05)	0.99 (0.94–1.04)	0.98 (0.94–1.03)	0.98 (0.93–1.03)
Q4	0.99 (0.96–1.03)	0.98 (0.94–1.03)	0.98 (0.93–1.02)	0.97 (0.92–1.03)
Q5 (highest inequality)	**0.92 (0.89–0.96)**	**0.91 (0.87–0.96)**	**0.92 (0.88–0.96)**	0.95 (0.90–1.01)
Percent unemployed				
Q1 (lowest %)	1.00 (ref)	1.00 (ref)	1.00 (ref)	1.00 (ref)
Q2	**1.08 (1.04–1.13)**	**1.09 (1.04–1.14)**	0.99 (0.95–1.04)	0.98 (0.93–1.04)
Q3	**1.12 (1.08–1.17)**	**1.13 (1.07–1.19)**	**1.06 (1.01–1.11)**	1.01 (0.95–1.06)
Q4	**1.21 (1.16–1.25)**	**1.22 (1.16–1.28)**	**1.09 (1.04–1.14)**	**1.08 (1.03–1.14)**
Q5 (highest %)	**1.31 (1.26–1.36)**	**1.29 (1.23–1.35)**	**1.14 (1.09–1.19)**	**1.08 (1.02–1.15)**
Percent uninsured				
Q1 (lowest %)	1.00 (ref)	1.00 (ref)	1.00 (ref)	1.00 (ref)
Q2	**1.13 (1.09–1.18)**	**1.10 (1.05–1.16)**	**1.11 (1.06–1.16)**	**1.08 (1.02–1.15)**
Q3	**1.23 (1.19–1.28)**	**1.23 (1.17–1.29)**	**1.13 (1.08–1.18)**	**1.13 (1.07–1.20)**
Q4	**1.29 (1.24–1.34)**	**1.27 (1.21–1.34)**	**1.18 (1.12–1.23)**	**1.17 (1.11–1.24)**
Q5 (highest %)	**1.36 (1.31–1.41)**	**1.37 (1.30–1.43)**	**1.16 (1.10–1.21)**	**1.12 (1.05–1.19)**
Percent without high school diploma				
Q1 (lowest %)	1.00 (ref)	1.00 (ref)	1.00 (ref)	1.00 (ref)
Q2	**1.12 (1.08–1.16)**	**1.10 (1.05–1.16)**	**1.12 (1.07–1.17)**	**1.08 (1.02–1.14)**
Q3	**1.23 (1.19–1.28)**	**1.23 (1.17–1.29)**	**1.20 (1.15–1.26)**	**1.14 (1.08–1.21)**
Q4	**1.36 (1.31–1.41)**	**1.36 (1.30–1.43)**	**1.24 (1.18–1.29)**	**1.18 (1.12–1.25)**
Q5 (highest %)	**1.41 (1.35–1.47)**	**1.38 (1.31–1.46)**	**1.27 (1.21–1.33)**	**1.18 (1.11–1.26)**

Analyses adjusted for age, race, ethnicity, marital status, primary payer, stage at cancer diagnosis, grade, subtype, year of diagnosis, and treatment status (surgery, chemotherapy, radiation for both California and New York cases; hormone and immunotherapy for New York cases only). Statistically significant associations are shown in bold. California patients were followed through December 2017; New York patients were followed through December 2018

In supplemental analyses of NYS data, we observed statistically significant interactions with race/ethnicity for associations of several socioeconomic variables with stage, subtype, OS, and CSS (Supplementary Tables 1–4). Stratified results for non-Hispanic White individuals were generally similar to those for all individuals combined, whereas associations for other racial and ethnic subgroups varied and were often not statistically significant. Exceptions included associations of median household income and percent without a high school diploma with poorer tumor differentiation among Hispanic individuals and associations of percent below the poverty line and percent without a high school diploma with higher odds of triple negative tumors in non-Hispanic Black individuals. In addition, several socioeconomic variables were associated with significantly poorer survival within strata of race and ethnicity, including associations of percent below the poverty line and income inequality with poorer survival in non-Hispanic Black individuals, associations of percent below the poverty line, median household income, percent uninsured, and percent without a high school diploma with poorer survival in non-Hispanic Asian/Pacific Islander individuals, and borderline statistically significant associations of percent below the poverty line and median household income with poorer survival in Hispanic individuals.

In supplemental NYS survival analyses we also observed statistically significant interactions with breast cancer subtype for the associations with percent below the poverty line, median household income, and percent uninsured, and a borderline significant interaction for the association with percent without a high school diploma (results not shown). In stratified analyses, associations of both poverty and median household income with OS and CSS were present among individuals with Luminal A and B subtypes but not among individuals with HER2-enriched or triple negative cancers. Similarly, associations with percent uninsured were present for all subtypes except triple negative cancers, and associations with percent without a high school diploma were present for the Luminal A and B subtypes only. Although the interaction between income inequality and tumor subtype was not statistically significant, the inverse associations with OS and CSS were present only for individuals with the Luminal A and B subtypes.

Pooled results from the California synthetic census tract data were consistent with results from the actual data in both direction and statistical significance (Supplementary Tables 5–8). However, the synthetic data tended to overestimate associations with stage and underestimate associations with grade, subtype, and survival due to its deterministic assumption for stage and random errors generated as part of the synthesis process, which led to attenuation effects for the other associations. Comparing the synthetic versus actual data, OR estimates for the highest versus lowest quintile were up to 21% higher for analyses of stage at diagnosis, up to 15% lower for analyses of tumor differentiation, and up to 16% lower for analyses of tumor subtype. Corresponding HR estimates were up to 21% lower for the synthetic versus actual data.

## Discussion

In this large analysis of 386,945 breast cancer patients from two diverse states, we observed that individuals who lived in more disadvantaged census tracts at diagnosis had poorer prognosis and survival, including more advanced stage at diagnosis, higher grade tumors, more aggressive subtypes, and poorer overall and cancer-specific survival. This was true for all socioeconomic variables except income inequality within a census tract, which was not clearly associated with poorer prognosis but was associated with slightly better survival. The results were generally consistent across the two states but somewhat stronger in California, particularly for the associations with tumor grade and survival.

These results confirm and build upon the results of multiple previous studies [2–4, 6, 10, 11, 14, 15, 17, 20, 21, 23, 24, 27, 29]. Our results for associations of neighborhood-level deprivation with breast cancer stage, aggressiveness, and survival were overall consistent with those reported in other, generally smaller, studies [2–4, 6, 10, 11, 14, 15, 17, 20, 21, 23, 24]. Fewer studies have examined measures of income inequality in relation to breast cancer outcomes. In three studies conducted in Brazil, high income inequality within a geographic area was associated with higher breast cancer mortality; however, these studies used 27 regions (Federative Units) with large populations, rather than smaller regions with less variability within each region [34–36]. To our knowledge, no U.S.-based studies of income inequality and breast cancer characteristics or outcomes have been published, although a study conducted in the U.S. reported that women diagnosed with breast cancer who lived in areas of high income inequality were more likely to be early recipients of gene expression profiling than women in areas of low income inequality [43]. This suggests that women in areas with higher income inequality may have earlier access to novel treatments, possibly contributing to improved survival.

In supplemental analyses for NYS, we observed statistically significant interactions with race and ethnicity in analyses of stage, tumor subtype, and survival, as well as statistically significant interactions with tumor subtype in survival analyses. In analyses stratified by race and ethnicity, associations were strongest in non-Hispanic White individuals, although some associations persisted among other racial and ethnic groups while other associations did not reach statistical significance. Previous studies have been mixed, with some reporting effect modification by race [2, 9, 25, 30, 44] and others reporting no differences by race and ethnicity [17, 23]. We did not confirm the stratified results in the California data because of limited access to the actual census tract data. Additional work is needed to explore the stratified associations in larger populations of non-White individuals. In survival analyses stratified by subtype, we observed statistically significant associations for multiple socioeconomic variables with survival among individuals with Luminal A and Luminal B tumors, but for individuals with HER2-enriched tumors only percent uninsured was associated with poorer survival and for individuals with triple negative tumors there were no statistically significant associations. 5-year relative survival is highest for women with Luminal A (95.6%) and Luminal B (91.8%) tumors, with poorer 5-year survival among women with HER2-enriched (86.5%) and triple negative tumors (78.4%) [45]. Our results suggest that census tract-level disadvantage is associated with poorer survival for tumor subtypes with a more favorable survival profile but not for subtypes with lower 5-year survival, possibly because other factors are stronger predictors of outcomes for the less favorable tumor subtypes. A previous analysis of SEER data similarly reported that breast cancer subtype modified the association between a census tract-level composite index of socioeconomic status and CSS, with stronger associations among the luminal and HER2-enriched subtypes compared to the triple negative subtype [25]. However, the prior study still observed statistically significant associations between lower socioeconomic index and poorer survival among triple negative cancers, potentially due to differences in the study populations or the exposure variables examined.

Our analysis provides a valuable use case for synthetic census tract data by allowing for comparison of results generated using synthetic and actual census tract data for California. Analysis results for the synthetic and actual census tract data were generally consistent, but the synthetic data tended to overestimate associations with stage and underestimate associations with grade, subtype, and survival. Although these differences were modest and did not change the interpretation of the results, our analysis supports prior assessments by Yu et al. [37] that synthetic data are best suited for initial analyses that should be validated against results from the actual data provided by an honest broker [46, 47]. Subsequent verification analyses, as conducted for this project, can help determine the use cases where analytical results on synthetic data are congruent with those from actual data versus where additional validation may be needed. Such projects also provide feedback for further improvement in synthetic data modeling. This approach allows researchers to conduct exploratory analyses at a finer geographic level than previously available while ensuring both patient confidentiality and yielding accurate results, although additional resources for the manual validation of each analysis may be required for any analyses that diverge from models underlying the synthetic data.

This study is one of the largest to examine census tract-level socioeconomic measures in relation to breast cancer prognosis and survival, and the first to examine associations of income inequality with breast cancer characteristics and outcomes in the U.S. Strengths include the large number of cases, small area socioeconomic data, and information on multiple potential confounders of interest. The consistency of the results across the two states strengthens the evidence for the associations observed. Where differences were observed in the results, they may have been due to residual confounding or differences in the quintile cut points or characteristics of the most disadvantaged census tracts in each state. In addition, the analyses of both synthetic and actual census tract data for California and the comparison of these results provides valuable information on use of synthetic census tract data in a real-world analysis.

Limitations of the analysis include the possibility of uncontrolled confounding by variables not available in the Registry data or residual confounding or effect modification by race, ethnicity, or other variables included in the analysis. Although NYS analyses indicated effect modification by race/ethnicity and tumor subtype, stratified analyses may have been underpowered to detect associations within smaller strata, and because of limited data access we did not run analyses stratified by race/ethnicity or tumor subtype on the actual California data to compare results from the two states. We examined each socioeconomic variable separately and did not adjust for other census tract-level variables, to avoid issues with collinearity due to highly statistically significant correlations between the socioeconomic variables of interest (all *p* < 0.0001, with Spearman correlation coefficients ranging from 0.50 to 0.83 for associations with all variables except Gini index of income inequality; results not shown). However, including multiple socioeconomic variables while accounting for multicollinearity could be informative. In addition, recent cases and follow-up data were not available for California, and as a result we limited the analysis for both states to cases diagnosed in 2006–2017 with a similar timeframe for survival but omitting California cases diagnosed in 2017. Further, some data of interest were not available, such as HER2 data for NYS cases diagnosed before 2010. We included data on primary payer at diagnosis as a covariate in analyses of stage at diagnosis and variables for receipt of treatment during initial course of treatment in survival analyses. These variables are subject to measurement error and may not accurately control for confounding. To help address possible underreporting, treatment variables were categorized as yes versus no/unknown, since the absence of treatment data can mean that the treatment was not administered or that it was administered but not reported. The treatment variables also do not take into account whether a certain treatment was indicated or recommended for a patient [48]. Census block group data would provide more specific socioeconomic information for patients residing within each geographic area; although census block group data were not available for this analysis, census tract is a finer geographic level than that used in many previous similar studies and is more likely to be representative of individuals within a census tract than measures at the county level. Finally, analyses of the synthetic and actual California census tract data were run on different machines, which may have introduced some minor differences in the results.

In summary, these results provide additional evidence that disadvantage at the census tract level is associated with poorer breast cancer prognosis and survival. In NYS, these associations were primarily observed among non-Hispanic White individuals but with some notable associations in other racial and ethnic subgroups. In both California and NYS, higher non-completion of high school was most strongly associated with advanced stage at diagnosis, and all census tract-level socioeconomic variables with the exception of income inequality were associated with poorer survival, particularly for tumor subtypes with a more favorable survival profile in NYS analyses. The California synthetic census tract data provided a reasonable approximation of the actual results, although the synthetic data overestimated some associations and underestimated others. The results indicate that synthetic data can be used to support exploratory analyses of small area geographic factors in relation to cancer prognosis and outcomes, with validation using actual data for confirmation of results. Although validation may limit efficiency and increase costs, it ensures valid results for publishing research findings. Overall, our results confirm previous studies showing poorer breast cancer prognosis and outcomes in more disadvantaged areas, and highlight the need for additional interventions to ensure early diagnosis and guideline-concordant care for breast cancer patients living in areas with high levels of disadvantage, to improve outcomes and survival for all breast cancer patients.

## Supplementary Information

Below is the link to the electronic supplementary material.

Supplementary file1 (DOCX 69 KB)

## Data Availability

The California data used for this project were restricted to authorized personnel per data use agreement. The authors do not have permission to share the data. The NYS data are available via a data request process. For more information please see the New York State Cancer Registry Data Release and Policy Manual available here: https://www.health.ny.gov/statistics/cancer/registry/docs/nyscr_data_release_and_policy_manual.pdf.
