# Supplementary material for: Census tract-level socioeconomic variables and breast cancer characteristics and outcomes in California and New York State
Source: Cancer Causes Control. 2026 Mar 19;37(4):62. doi: 10.1007/s10552-026-02152-1 (PMC13002732; doi:10.1007/s10552-026-02152-1)
Supplement: Supplementary file 1 — Supplementary file1 (DOCX 69 KB) [file 10552_2026_2152_MOESM1_ESM.docx]

| Supplementary Table 1. Multivariable-adjusted odds ratios (OR) and 95% confidence intervals (CI) for associations of census tract-level socioeconomic variables and breast cancer stage at diagnosis for invasive breast cancer cases diagnosed in 2006-2017 in New York State, stratified by race/ethnicity* | | | | | | | | | |
| --- | --- | --- | --- | --- | --- | --- | --- | --- | --- |
| Census tract-level exposure variables | **Race/ethnicity** | | | | | | | |  |
|  | **Non-Hispanic White** | | **Non-Hispanic Black** | | **Non-Hispanic Asian/**  **Pacific Islander** | | **Hispanic** | |  |
|  | **Regional**^†^ | **Distant**^†^ | **Regional**^†^ | **Distant**^†^ | **Regional**^†^ | **Distant**^†^ | **Regional**^†^ | **Distant**^†^ |  |
| Percent below poverty line (*P*-interaction^‡^ with race/ethnicity = 0.002) | | | | | | | | |  |
| Q1 (lowest %) | 1.00 (ref) | 1.00 (ref) | 1.00 (ref) | 1.00 (ref) | 1.00 (ref) | 1.00 (ref) | 1.00 (ref) | 1.00 (ref) |  |
| Q2 | **1.06 (1.02-1.11)** | 1.05 (0.96-1.14) | 0.96 (0.81-1.13) | 0.85 (0.65-1.12) | 0.97 (0.81-1.16) | 1.24 (0.79-1.96) | 1.07 (0.90-1.27) | 1.13 (0.79-1.61) |  |
| Q3 | **1.13 (1.08-1.18)** | **1.15 (1.06-1.25)** | 0.88 (0.76-1.02) | 0.95 (0.74-1.20) | 1.03 (0.87-1.22) | 1.27 (0.82-1.97) | 0.95 (0.81-1.12) | 0.98 (0.70-1.38) |  |
| Q4 | **1.16 (1.11-1.22)** | **1.25 (1.14-1.36)** | 0.99 (0.86-1.14) | 0.92 (0.73-1.17) | 0.93 (0.80-1.09) | 1.11 (0.73-1.69) | 1.10 (0.94-1.28) | 1.05 (0.76-1.43) |  |
| Q5 (highest %) | **1.19 (1.12-1.25)** | **1.37 (1.24-1.52)** | 1.08 (0.94-1.24) | 0.98 (0.79-1.23) | 1.06 (0.91-1.25) | 1.25 (0.82-1.91) | 1.11 (0.97-1.28) | 1.03 (0.76-1.38) |  |
| Median household income (*P*-interaction^‡^ with race/ethnicity = 0.04) | | | | | | | | |  |
| Q1 (highest income) | 1.00 (ref) | 1.00 (ref) | 1.00 (ref) | 1.00 (ref) | 1.00 (ref) | 1.00 (ref) | 1.00 (ref) | 1.00 (ref) |  |
| Q2 | **1.08 (1.03-1.13)** | **1.10 (1.01-1.20)** | 1.07 (0.91-1.25) | 1.13 (0.88-1.46) | 1.14 (0.97-1.34) | 1.32 (0.90-1.92) | 0.99 (0.85-1.16) | 1.21 (0.88-1.66) |  |
| Q3 | **1.15 (1.10-1.20)** | **1.16 (1.07-1.27)** | 1.15 (0.99-1.35) | 1.07 (0.83-1.37) | 1.11 (0.94-1.30) | 1.25 (0.86-1.82) | 1.13 (0.97-1.31) | 0.88 (0.64-1.21) |  |
| Q4 | **1.15 (1.10-1.20)** | **1.18 (1.08-1.29)** | 1.10 (0.95-1.28) | 1.07 (0.84-1.36) | 1.00 (0.85-1.17) | 1.10 (0.76-1.60) | 1.07 (0.93-1.23) | 1.02 (0.75-1.39) |  |
| Q5 (lowest income) | **1.21 (1.14-1.27)** | **1.40 (1.26-1.54)** | **1.20 (1.04-1.38)** | 1.05 (0.84-1.33) | 1.06 (0.91-1.24) | 1.27 (0.88-1.82) | 1.13 (0.99-1.29) | 1.14 (0.86-1.50) |  |
| Gini index of income inequality (*P*-interaction^‡^ with race/ethnicity = 0.06) | | | | | | | | |  |
| Q1 (lowest inequality) | 1.00 (ref) | 1.00 (ref) | 1.00 (ref) | 1.00 (ref) | 1.00 (ref) | 1.00 (ref) | 1.00 (ref) | 1.00 (ref) |  |
| Q2 | 1.02 (0.98-1.07) | 0.95 (0.87-1.03) | 0.92 (0.82-1.04) | 0.97 (0.81-1.17) | 1.13 (0.94-1.36) | 0.97 (0.62-1.52) | 1.05 (0.91-1.21) | 0.98 (0.72-1.34) |  |
| Q3 | 1.00 (0.96-1.05) | 0.95 (0.87-1.04) | 0.92 (0.82-1.03) | **0.79 (0.66-0.94)** | 1.02 (0.86-1.22) | 0.70 (0.45-1.09) | 1.12 (0.98-1.29) | 1.00 (0.75-1.34) |  |
| Q4 | 1.00 (0.95-1.05) | 0.96 (0.88-1.05) | 0.97 (0.88-1.08) | 0.88 (0.75-1.04) | 0.98 (0.82-1.16) | 0.91 (0.60-1.36) | 0.97 (0.86-1.10) | 1.17 (0.90-1.53) |  |
| Q5 (highest inequality) | 0.96 (0.91-1.00) | **0.83 (0.75-0.91)** | 1.00 (0.90-1.10) | 1.00 (0.86-1.18) | 0.94 (0.79-1.12) | 0.81 (0.53-1.23) | 1.00 (0.88-1.13) | 1.09 (0.83-1.41) |  |
| Percent unemployed (*P*-interaction^‡^ with race/ethnicity = 0.04) | | | | | | | | |  |
| Q1 (lowest %) | 1.00 (ref) | 1.00 (ref) | 1.00 (ref) | 1.00 (ref) | 1.00 (ref) | 1.00 (ref) | 1.00 (ref) | 1.00 (ref) |  |
| Q2 | 1.03 (0.99-1.08) | **1.17 (1.07-1.27)** | 0.86 (0.73-1.02) | 0.90 (0.69-1.17) | 1.11 (0.94-1.30) | 1.14 (0.75-1.72) | 1.14 (0.97-1.33) | 1.27 (0.92-1.76) |  |
| Q3 | **1.06 (1.01-1.10)** | **1.20 (1.11-1.31)** | 0.92 (0.79-1.07) | 0.91 (0.71-1.17) | 0.98 (0.84-1.15) | 1.12 (0.75-1.66) | 1.14 (0.98-1.32) | 1.28 (0.94-1.75) |  |
| Q4 | **1.12 (1.08-1.18)** | **1.26 (1.15-1.37)** | 1.02 (0.88-1.17) | 0.89 (0.71-1.12) | 0.94 (0.81-1.10) | 1.15 (0.79-1.68) | **1.20 (1.04-1.37)** | 1.19 (0.89-1.59) |  |
| Q5 (highest %) | **1.13 (1.08-1.20)** | **1.40 (1.27-1.54)** | 1.03 (0.90-1.18) | 0.93 (0.75-1.16) | 1.12 (0.96-1.30) | 1.15 (0.78-1.70) | **1.20 (1.05-1.36)** | 1.22 (0.93-1.60) |  |
| Percent uninsured (*P*-interaction^‡^ with race/ethnicity = 0.001) | | | | | | | | |  |
| Q1 (lowest %) | 1.00 (ref) | 1.00 (ref) | 1.00 (ref) | 1.00 (ref) | 1.00 (ref) | 1.00 (ref) | 1.00 (ref) | 1.00 (ref) |  |
| Q2 | 1.04 (1.00-1.08) | **1.18 (1.08-1.28)** | 1.20 (0.98-1.46) | 1.41 (1.00-1.98) | 1.14 (0.95-1.38) | 0.91 (0.57-1.45) | 0.99 (0.82-1.19) | 0.88 (0.61-1.27) |  |
| Q3 | **1.11 (1.06-1.16)** | **1.25 (1.15-1.37)** | 1.13 (0.95-1.36) | 1.27 (0.92-1.74) | 1.10 (0.91-1.33) | 0.82 (0.51-1.32) | 1.06 (0.89-1.26) | 0.80 (0.56-1.13) |  |
| Q4 | **1.20 (1.14-1.25)** | **1.36 (1.25-1.49)** | 1.14 (0.96-1.36) | 1.34 (0.99-1.82) | 1.18 (0.99-1.41) | 0.93 (0.60-1.43) | 1.08 (0.91-1.27) | 0.91 (0.66-1.25) |  |
| Q5 (highest %) | **1.21 (1.14-1.27)** | **1.54 (1.40-1.71)** | 1.19 (1.00-1.41) | 1.32 (0.97-1.78) | 1.08 (0.92-1.26) | 0.94 (0.64-1.38) | 1.16 (1.00-1.36) | 0.87 (0.64-1.18) |  |
| Percent without high school diploma (*P*-interaction^‡^ with race/ethnicity = 0.12) | | | | | | | | |  |
| Q1 (lowest %) | 1.00 (ref) | 1.00 (ref) | 1.00 (ref) | 1.00 (ref) | 1.00 (ref) | 1.00 (ref) | 1.00 (ref) | 1.00 (ref) |  |
| Q2 | **1.09 (1.04-1.14)** | **1.22 (1.12-1.33)** | 1.18 (0.96-1.45) | 0.89 (0.63-1.25) | 1.06 (0.87-1.27) | 1.08 (0.67-1.73) | 1.16 (0.96-1.41) | 1.16 (0.78-1.72) |  |
| Q3 | **1.13 (1.08-1.18)** | **1.31 (1.20-1.43)** | 1.13 (0.94-1.37) | 1.03 (0.76-1.40) | 1.09 (0.91-1.32) | 1.33 (0.85-2.09) | 1.08 (0.90-1.30) | 1.04 (0.71-1.52) |  |
| Q4 | **1.20 (1.14-1.25)** | **1.47 (1.35-1.61)** | **1.23 (1.03-1.47)** | 1.04 (0.78-1.39) | 1.06 (0.89-1.25) | 1.49 (1.00-2.23) | 1.15 (0.97-1.35) | 1.25 (0.88-1.76) |  |
| Q5 (highest %) | **1.26 (1.18-1.33)** | **1.73 (1.55-1.92)** | **1.28 (1.07-1.53)** | 1.05 (0.79-1.40) | 1.12 (0.96-1.31) | 1.29 (0.88-1.90) | **1.19 (1.02-1.38)** | 1.08 (0.78-1.49) |  |

*Analyses adjusted for age, marital status, primary payer, and year of diagnosis.

^†^Stage at diagnosis compared to local stage disease; estimates for unknown stage omitted from table.

^‡^*P*-value for interaction from likelihood ratio test comparing models with and without interaction between exposure variable and race/ethnicity.

| Supplementary Table 2. Multivariable-adjusted odds ratios (OR) and 95% confidence intervals (CI) for associations of census tract-level socioeconomic variables and breast tumor differentiation for invasive breast cancer cases diagnosed in 2006-2017 in New York State, stratified by race/ethnicity* | | | | | | | | |
| --- | --- | --- | --- | --- | --- | --- | --- | --- |
| Census tract-level exposure variables | **Race/ethnicity** | | | | | | | |
|  | **Non-Hispanic White** | | **Non-Hispanic Black** | | **Non-Hispanic Asian/**  **Pacific Islander** | | **Hispanic** | |
|  | **Poorly/un-**  **differentiated**^†^ | **Unknown**^†^ | **Poorly/un-**  **differentiated**^†^ | **Unknown**^†^ | **Poorly/un-**  **differentiated**^†^ | **Unknown**^†^ | **Poorly/un-**  **differentiated**^†^ | **Unknown**^†^ |
| Percent below poverty line (*P*-interaction^‡^ with race/ethnicity = 0.73) | | | | | | | | |
| Q1 (lowest %) | 1.00 (ref) | 1.00 (ref) | 1.00 (ref) | 1.00 (ref) | 1.00 (ref) | 1.00 (ref) | 1.00 (ref) | 1.00 (ref) |
| Q2 | 1.01 (0.96-1.05) | 1.01 (0.93-1.10) | 1.01 (0.87-1.18) | 1.07 (0.82-1.40) | 1.00 (0.84-1.20) | 1.05 (0.78-1.41) | 0.95 (0.80-1.13) | 1.14 (0.85-1.52) |
| Q3 | **1.06 (1.01-1.10)** | 1.04 (0.96-1.13) | 1.00 (0.87-1.14) | 1.00 (0.78-1.27) | 1.09 (0.92-1.28) | 1.01 (0.76-1.34) | 1.03 (0.88-1.21) | 1.26 (0.96-1.65) |
| Q4 | **1.08 (1.03-1.13)** | **1.14 (1.05-1.24)** | 0.98 (0.86-1.12) | 1.06 (0.84-1.34) | 1.05 (0.90-1.22) | 1.21 (0.93-1.57) | **1.25 (1.08-1.45)** | **1.34 (1.04-1.74)** |
| Q5 (highest %) | **1.14 (1.08-1.20)** | **1.29 (1.18-1.43)** | 1.08 (0.95-1.22) | 1.20 (0.96-1.50) | 1.08 (0.92-1.26) | **1.39 (1.07-1.80)** | 1.13 (0.99-1.30) | **1.46 (1.15-1.85)** |
| Median household income (*P*-interaction^‡^ with race/ethnicity = 0.12) | | | | | | | | |
| Q1 (highest income) | 1.00 (ref) | 1.00 (ref) | 1.00 (ref) | 1.00 (ref) | 1.00 (ref) | 1.00 (ref) | 1.00 (ref) | 1.00 (ref) |
| Q2 | 1.02 (0.98-1.07) | **0.91 (0.84-0.99)** | 1.09 (0.94-1.26) | 0.82 (0.64-1.05) | 0.98 (0.83-1.15) | 0.88 (0.67-1.14) | 1.03 (0.89-1.20) | **1.38 (1.07-1.78)** |
| Q3 | 1.03 (0.99-1.08) | **0.87 (0.80-0.95)** | 1.09 (0.94-1.25) | 0.94 (0.74-1.19) | 1.15 (0.99-1.35) | 0.81 (0.62-1.06) | 1.09 (0.94-1.27) | 1.20 (0.93-1.54) |
| Q4 | **1.10 (1.05-1.15)** | **0.87 (0.80-0.95)** | 1.15 (1.00-1.32) | 0.97 (0.77-1.23) | 1.00 (0.86-1.17) | 0.98 (0.76-1.25) | **1.26 (1.09-1.45)** | **1.29 (1.01-1.65)** |
| Q5 (lowest income) | **1.10 (1.05-1.16)** | 1.07 (0.97-1.17) | **1.16 (1.02-1.32)** | 1.01 (0.81-1.25) | 1.08 (0.93-1.26) | **1.28 (1.01-1.63)** | **1.14 (1.01-1.30)** | **1.43 (1.15-1.78)** |
| Gini index of income inequality (*P*-interaction^‡^ with race/ethnicity = 0.27) | | | | | | | | |
| Q1 (lowest inequality) | 1.00 (ref) | 1.00 (ref) | 1.00 (ref) | 1.00 (ref) | 1.00 (ref) | 1.00 (ref) | 1.00 (ref) | 1.00 (ref) |
| Q2 | 1.02 (0.97-1.06) | 1.06 (0.97-1.15) | 1.04 (0.94-1.16) | 0.98 (0.82-1.18) | 1.07 (0.89-1.29) | **1.44 (1.05-1.98)** | 1.14 (0.99-1.32) | 1.08 (0.85-1.39) |
| Q3 | 1.02 (0.98-1.07) | **1.15 (1.06-1.26)** | 1.03 (0.93-1.14) | 0.92 (0.77-1.11) | 1.00 (0.84-1.19) | 1.21 (0.89-1.65) | 1.15 (1.00-1.31) | 1.25 (1.00-1.57) |
| Q4 | **1.09 (1.04-1.15)** | **1.33 (1.22-1.45)** | 1.06 (0.97-1.17) | 1.13 (0.96-1.33) | 1.07 (0.91-1.27) | **1.39 (1.04-1.86)** | 1.13 (0.99-1.28) | **1.25 (1.01-1.54)** |
| Q5 (highest inequality) | **1.06 (1.01-1.12)** | **1.41 (1.29-1.53)** | **1.11 (1.01-1.21)** | 1.14 (0.97-1.34) | 1.10 (0.92-1.30) | **1.52 (1.13-2.05)** | 1.10 (0.97-1.24) | **1.28 (1.04-1.58)** |
| Percent unemployed (*P*-interaction^‡^ with race/ethnicity = 0.13) | | | | | | | | |
| Q1 (lowest %) | 1.00 (ref) | 1.00 (ref) | 1.00 (ref) | 1.00 (ref) | 1.00 (ref) | 1.00 (ref) | 1.00 (ref) | 1.00 (ref) |
| Q2 | 1.03 (0.99-1.08) | **1.11 (1.02-1.21)** | 1.00 (0.85-1.16) | 0.78 (0.59-1.02) | 0.93 (0.79-1.09) | 1.05 (0.80-1.37) | 1.03 (0.89-1.20) | 1.20 (0.92-1.55) |
| Q3 | 1.04 (1.00-1.09) | **1.11 (1.02-1.21)** | 0.97 (0.84-1.12) | 0.96 (0.75-1.22) | 0.86 (0.73-1.00) | 1.05 (0.81-1.35) | 0.92 (0.79-1.06) | 1.19 (0.93-1.52) |
| Q4 | **1.07 (1.02-1.12)** | **1.23 (1.13-1.34)** | 1.12 (0.98-1.28) | 0.96 (0.77-1.21) | 0.88 (0.76-1.03) | 1.15 (0.91-1.47) | 1.05 (0.91-1.20) | 1.19 (0.94-1.50) |
| Q5 (highest %) | **1.10 (1.04-1.16)** | **1.24 (1.13-1.37)** | 1.06 (0.93-1.20) | 1.01 (0.82-1.25) | **0.83 (0.71-0.97)** | 1.15 (0.90-1.47) | 1.00 (0.88-1.13) | **1.32 (1.07-1.64)** |
| Percent uninsured (*P*-interaction^‡^ with race/ethnicity = 0.81) | | | | | | | | |
| Q1 (lowest %) | 1.00 (ref) | 1.00 (ref) | 1.00 (ref) | 1.00 (ref) | 1.00 (ref) | 1.00 (ref) | 1.00 (ref) | 1.00 (ref) |
| Q2 | 0.99 (0.95-1.04) | 0.96 (0.89-1.04) | 1.06 (0.88-1.27) | 1.08 (0.79-1.47) | 0.99 (0.82-1.19) | 1.08 (0.80-1.47) | 0.98 (0.82-1.18) | 0.95 (0.70-1.28) |
| Q3 | 1.03 (0.99-1.08) | 1.08 (0.99-1.17) | 0.98 (0.83-1.15) | 0.95 (0.71-1.26) | 1.06 (0.88-1.27) | 1.01 (0.74-1.39) | 1.02 (0.85-1.21) | 1.05 (0.79-1.40) |
| Q4 | **1.11 (1.06-1.16)** | **1.13 (1.04-1.23)** | 0.97 (0.83-1.14) | 0.96 (0.73-1.26) | 1.06 (0.89-1.26) | 1.11 (0.84-1.48) | 1.14 (0.97-1.34) | 1.09 (0.83-1.41) |
| Q5 (highest %) | **1.12 (1.06-1.18)** | **1.31 (1.19-1.44)** | 1.03 (0.88-1.20) | 1.06 (0.81-1.39) | 1.06 (0.91-1.23) | 1.20 (0.94-1.55) | 1.13 (0.97-1.32) | 1.19 (0.93-1.53) |
|  |  |  |  |  |  |  |  |  |
| Percent without high school diploma (*P*-interaction^‡^ with race/ethnicity = 0.27) | | | | | | | | |
| Q1 (lowest %) | 1.00 (ref) | 1.00 (ref) | 1.00 (ref) | 1.00 (ref) | 1.00 (ref) | 1.00 (ref) | 1.00 (ref) | 1.00 (ref) |
| Q2 | 1.03 (0.98-1.07) | 1.00 (0.93-1.09) | 1.09 (0.90-1.32) | 1.03 (0.74-1.44) | 0.94 (0.78-1.13) | 0.94 (0.69-1.27) | 1.16 (0.96-1.41) | **1.42 (1.02-1.97)** |
| Q3 | 1.04 (0.99-1.08) | 1.02 (0.94-1.11) | 1.11 (0.94-1.32) | 0.99 (0.73-1.34) | 0.97 (0.81-1.17) | 0.87 (0.64-1.19) | 1.14 (0.95-1.36) | **1.46 (1.07-2.00)** |
| Q4 | **1.09 (1.04-1.14)** | **1.19 (1.09-1.29)** | 1.12 (0.96-1.32) | 1.07 (0.80-1.42) | 1.15 (0.97-1.36) | 0.94 (0.72-1.24) | **1.32 (1.12-1.55)** | **1.47 (1.10-1.96)** |
| Q5 (highest %) | **1.15 (1.08-1.22)** | **1.42 (1.29-1.57)** | 1.11 (0.95-1.31) | 1.21 (0.92-1.60) | 1.02 (0.88-1.19) | 1.19 (0.93-1.52) | **1.28 (1.11-1.49)** | **1.64 (1.26-2.13)** |

*Analyses adjusted for age and year of diagnosis.

^†^Reference category for tumor differentiation is well/moderately well differentiated.

^‡^*P*-value for interaction from likelihood ratio test comparing models with and without interaction between exposure variable and race/ethnicity.

| Supplementary Table 3. Multivariable-adjusted odds ratios (OR) and 95% confidence intervals (CI) for associations of census tract-level socioeconomic variables and breast tumor subtype for invasive breast cancer cases diagnosed in 2010-2017 in New York State, stratified by race/ethnicity* | | | | | | | | | | | | |
| --- | --- | --- | --- | --- | --- | --- | --- | --- | --- | --- | --- | --- |
| Census tract-level exposure variables | **Race/ethnicity** | | | | | | | | | | |  |
|  | **Non-Hispanic White** | | | | | | **Non-Hispanic Black** | | | | |  |
|  | **HR+/HER2+**  **subtype**^†^ | | **HR-/HER2+ subtype**^†^ | | **HR-/HER2- subtype**^†^ | | **HR+/HER2+ subtype**^†^ | | **HR-/HER2+ subtype**^†^ | | **HR-/HER2- subtype**^†^ |  |
| Percent below poverty line (*P*-interaction^‡^ with race/ethnicity = 0.13) | | | | | | | | | | | |  |
| Q1 (lowest %) | 1.00 (ref) | | 1.00 (ref) | | 1.00 (ref) | | 1.00 (ref) | | 1.00 (ref) | | 1.00 (ref) |  |
| Q2 | 1.01 (0.94-1.09) | | 1.03 (0.92-1.16) | | 1.06 (0.98-1.16) | | 1.09 (0.82-1.44) | | 0.89 (0.61-1.30) | | 1.06 (0.84-1.34) |  |
| Q3 | 1.05 (0.97-1.13) | | 1.10 (0.98-1.24) | | 1.09 (1.00-1.19) | | 0.97 (0.75-1.26) | | 1.03 (0.74-1.44) | | 1.07 (0.87-1.33) |  |
| Q4 | **1.13 (1.04-1.22)** | | 1.08 (0.95-1.22) | | **1.17 (1.08-1.28)** | | 1.17 (0.91-1.50) | | 1.04 (0.75-1.43) | | 1.12 (0.91-1.38) |  |
| Q5 (highest %) | 1.09 (0.99-1.21) | | **1.21 (1.04-1.40)** | | **1.23 (1.11-1.36)** | | 1.11 (0.88-1.40) | | 1.03 (0.76-1.39) | | **1.28 (1.05-1.55)** |  |
| Median household income (*P*-interaction^‡^ with race/ethnicity = 0.008) | | | | | | | | | | | |  |
| Q1 (highest income) | 1.00 (ref) | | 1.00 (ref) | | 1.00 (ref) | | 1.00 (ref) | | 1.00 (ref) | | 1.00 (ref) |  |
| Q2 | 1.03 (0.95-1.11) | | 1.11 (0.99-1.25) | | **1.20 (1.10-1.30)** | | 1.02 (0.77-1.33) | | 1.08 (0.76-1.53) | | 0.98 (0.78-1.22) |  |
| Q3 | 1.05 (0.97-1.14) | | **1.17 (1.03-1.31)** | | **1.25 (1.15-1.36)** | | 1.10 (0.84-1.43) | | 1.03 (0.73-1.45) | | 0.93 (0.75-1.16) |  |
| Q4 | 1.05 (0.97-1.14) | | **1.17 (1.03-1.32)** | | **1.32 (1.21-1.44)** | | 1.14 (0.88-1.48) | | 0.97 (0.69-1.37) | | 1.13 (0.92-1.39) |  |
| Q5 (lowest income) | 1.06 (0.96-1.17) | | **1.34 (1.16-1.54)** | | **1.42 (1.28-1.56)** | | 1.09 (0.85-1.39) | | 0.99 (0.72-1.35) | | 1.13 (0.93-1.38) |  |
| Gini index of income inequality (*P*-interaction^‡^ with race/ethnicity = 0.004) | | | | | | | | | | | |  |
| Q1 (lowest inequality) | 1.00 (ref) | | 1.00 (ref) | | 1.00 (ref) | | 1.00 (ref) | | 1.00 (ref) | | 1.00 (ref) |  |
| Q2 | **1.09 (1.01-1.18)** | | 0.98 (0.87-1.10) | | 1.00 (0.92-1.09) | | **1.30 (1.07-1.57)** | | 1.06 (0.82-1.36) | | 1.04 (0.89-1.23) |  |
| Q3 | 1.03 (0.95-1.12) | | 0.89 (0.79-1.01) | | 0.97 (0.89-1.06) | | 1.12 (0.93-1.36) | | **0.75 (0.58-0.97)** | | 1.01 (0.86-1.18) |  |
| Q4 | 1.09 (1.00-1.18) | | 0.96 (0.85-1.09) | | 1.00 (0.91-1.09) | | 1.08 (0.91-1.30) | | 0.89 (0.71-1.12) | | **1.17 (1.02-1.36)** |  |
| Q5 (highest inequality) | 1.05 (0.96-1.15) | | 0.90 (0.79-1.02) | | **0.85 (0.77-0.93)** | | 1.11 (0.93-1.32) | | 0.99 (0.79-1.24) | | 1.16 (1.00-1.34) |  |
| Percent unemployed (*P*-interaction^‡^ with race/ethnicity = 0.36) | | | | | | | | | | | |  |
| Q1 (lowest %) | 1.00 (ref) | | 1.00 (ref) | | 1.00 (ref) | | 1.00 (ref) | | 1.00 (ref) | | 1.00 (ref) |  |
| Q2 | 1.08 (1.00-1.17) | | **1.16 (1.03-1.30)** | | 1.08 (0.99-1.17) | | 0.86 (0.65-1.15) | | 0.87 (0.60-1.28) | | 0.88 (0.69-1.13) |  |
| Q3 | 1.06 (0.98-1.15) | | 1.11 (0.99-1.25) | | 1.09 (1.00-1.18) | | 0.99 (0.76-1.28) | | 1.00 (0.71-1.42) | | 1.14 (0.92-1.43) |  |
| Q4 | 1.05 (0.97-1.14) | | **1.19 (1.05-1.34)** | | **1.10 (1.01-1.20)** | | 1.02 (0.80-1.30) | | 1.02 (0.74-1.42) | | 1.19 (0.97-1.47) |  |
| Q5 (highest %) | **1.13 (1.02-1.24)** | | **1.20 (1.04-1.39)** | | **1.23 (1.11-1.36)** | | 0.97 (0.77-1.22) | | 0.95 (0.70-1.30) | | 1.12 (0.92-1.37) |  |
| Percent uninsured (*P*-interaction^‡^ with race/ethnicity = 0.04) | | | | | | | | | | | |  |
| Q1 (lowest %) | 1.00 (ref) | | 1.00 (ref) | | 1.00 (ref) | | 1.00 (ref) | | 1.00 (ref) | | 1.00 (ref) |  |
| Q2 | 1.01 (0.93-1.09) | | 1.02 (0.91-1.14) | | 1.06 (0.97-1.15) | | 0.75 (0.54-1.05) | | 1.01 (0.64-1.59) | | 0.85 (0.64-1.11) |  |
| Q3 | 1.01 (0.93-1.10) | | 1.09 (0.97-1.22) | | **1.14 (1.05-1.24)** | | 0.87 (0.64-1.17) | | 0.93 (0.61-1.41) | | 0.90 (0.70-1.15) |  |
| Q4 | 1.09 (1.00-1.18) | | **1.20 (1.06-1.36)** | | **1.18 (1.08-1.29)** | | 0.87 (0.66-1.16) | | 0.95 (0.63-1.41) | | 0.91 (0.72-1.15) |  |
| Q5 (highest %) | **1.18 (1.07-1.30)** | | **1.17 (1.01-1.35)** | | **1.15 (1.04-1.28)** | | 0.82 (0.62-1.09) | | 1.01 (0.68-1.50) | | 0.88 (0.69-1.11) |  |
|  |  |  | |  | |  |  |  | |  | |  |
| Percent without high school diploma (*P*-interaction^‡^ with race/ethnicity = 0.004) | | | | | | | | | | | |  |
| Q1 (lowest %) | 1.00 (ref) | | 1.00 (ref) | | 1.00 (ref) | | 1.00 (ref) | | 1.00 (ref) | | 1.00 (ref) |  |
| Q2 | 0.98 (0.91-1.06) | | 1.03 (0.92-1.16) | | **1.12 (1.03-1.21)** | | 1.06 (0.74-1.52) | | 0.80 (0.50-1.29) | | 1.05 (0.77-1.43) |  |
| Q3 | 1.02 (0.95-1.11) | | **1.18 (1.05-1.32)** | | **1.15 (1.06-1.25)** | | 1.07 (0.77-1.48) | | 0.99 (0.65-1.49) | | 1.25 (0.95-1.65) |  |
| Q4 | **1.10 (1.01-1.19)** | | 1.10 (0.97-1.24) | | **1.19 (1.09-1.30)** | | 1.19 (0.87-1.61) | | 1.00 (0.68-1.48) | | 1.27 (0.98-1.65) |  |
| Q5 (highest %) | 1.08 (0.97-1.21) | | **1.26 (1.07-1.47)** | | **1.36 (1.22-1.52)** | | 1.12 (0.83-1.51) | | 0.98 (0.67-1.44) | | **1.34 (1.03-1.73)** |  |

*Analyses adjusted for age and year of diagnosis. Estimates are presented for non-Hispanic Black and non-Hispanic White individuals only due to instability of stratified estimates for other categories of race and ethnicity.

^†^Reference category for tumor subtype is HR+/HER2-. Cases diagnosed before 2010 were excluded and cases with missing or unknown HR or HER2 status are omitted from table.

^‡^*P*-value for interaction from likelihood ratio test comparing models with and without interaction between exposure variable and race/ethnicity.

| Supplementary Table 4. Multivariable-adjusted hazard ratios (HR) and 95% confidence intervals (CI) for associations of census tract-level socioeconomic variables and overall and cancer-specific survival for invasive breast cancer cases diagnosed in 2006-2017 in New York State, stratified by race/ethnicity* | | | | | | | | | |
| --- | --- | --- | --- | --- | --- | --- | --- | --- | --- |
| Census tract-level exposure variables | **Race/ethnicity** | | | | | | | |  |
|  | **Non-Hispanic White** | | **Non-Hispanic Black** | | **Non-Hispanic Asian/**  **Pacific Islander** | | **Hispanic** | |  |
|  | **Overall**  **survival** | **Cancer-specific** | **Overall survival** | **Cancer-specific** | **Overall survival** | **Cancer-specific** | **Overall survival** | **Cancer-specific** |  |
| Percent below poverty line (*P*-interaction^†^ with race/ethnicity = 0.47 for overall and 0.37 for cancer-specific survival) | | | | | | | | |  |
| Q1 (lowest %) | 1.00 (ref) | 1.00 (ref) | 1.00 (ref) | 1.00 (ref) | 1.00 (ref) | 1.00 (ref) | 1.00 (ref) | 1.00 (ref) |  |
| Q2 | 1.05 (1.00-1.10) | 1.05 (1.00-1.10) | 1.06 (0.90-1.23) | 1.05 (0.89-1.23) | 1.17 (0.90-1.51) | 1.15 (0.89-1.50) | 1.18 (0.96-1.45) | 1.21 (0.98-1.49) |  |
| Q3 | **1.12 (1.07-1.17)** | **1.12 (1.07-1.18)** | 1.03 (0.90-1.18) | 1.04 (0.90-1.19) | 1.11 (0.85-1.45) | 1.09 (0.83-1.43) | 1.01 (0.83-1.23) | 1.02 (0.84-1.24) |  |
| Q4 | **1.15 (1.09-1.21)** | **1.15 (1.09-1.21)** | 1.09 (0.94-1.25) | 1.08 (0.94-1.24) | 1.24 (0.98-1.58) | 1.24 (0.97-1.58) | 1.03 (0.86-1.25) | 1.06 (0.88-1.28) |  |
| Q5 (highest %) | **1.23 (1.15-1.31)** | **1.23 (1.15-1.31)** | 1.14 (1.00-1.30) | **1.15 (1.01-1.32)** | **1.30 (1.03-1.65)** | **1.29 (1.01-1.65)** | 1.17 (0.99-1.39) | 1.18 (1.00-1.40) |  |
| Median household income (*P*-interaction^†^ with race/ethnicity = 0.002 for overall and 0.03 for cancer-specific survival) | | | | | | | | |  |
| Q1 (highest income) | 1.00 (ref) | 1.00 (ref) | 1.00 (ref) | 1.00 (ref) | 1.00 (ref) | 1.00 (ref) | 1.00 (ref) | 1.00 (ref) |  |
| Q2 | **1.11 (1.06-1.16)** | **1.11 (1.06-1.16)** | 0.96 (0.83-1.11) | 0.95 (0.82-1.10) | 1.15 (0.91-1.46) | 1.18 (0.93-1.50) | 1.04 (0.87-1.25) | 1.06 (0.88-1.27) |  |
| Q3 | **1.23 (1.18-1.30)** | **1.24 (1.18-1.30)** | 0.97 (0.84-1.12) | 0.96 (0.83-1.11) | 1.22 (0.95-1.57) | 1.24 (0.96-1.60) | 1.07 (0.90-1.28) | 1.08 (0.90-1.29) |  |
| Q4 | **1.28 (1.22-1.35)** | **1.28 (1.22-1.35)** | 0.95 (0.83-1.09) | 0.95 (0.82-1.09) | 1.18 (0.93-1.49) | 1.20 (0.94-1.52) | 1.02 (0.85-1.22) | 1.02 (0.85-1.22) |  |
| Q5 (lowest income) | **1.29 (1.21-1.37)** | **1.29 (1.21-1.37)** | 1.04 (0.91-1.18) | 1.04 (0.91-1.19) | **1.43 (1.14-1.79)** | **1.44 (1.14-1.82)** | 1.17 (1.00-1.37) | **1.18 (1.01-1.38)** |  |
| Gini index of income inequality (*P*-interaction^†^ with race/ethnicity < 0.001 for both overall and cancer-specific survival) | | | | | | | | |  |
| Q1 (lowest inequality) | 1.00 (ref) | 1.00 (ref) | 1.00 (ref) | 1.00 (ref) | 1.00 (ref) | 1.00 (ref) | 1.00 (ref) | 1.00 (ref) |  |
| Q2 | 0.98 (0.94-1.03) | 0.98 (0.93-1.03) | 1.07 (0.96-1.19) | 1.07 (0.96-1.19) | 0.86 (0.66-1.14) | 0.87 (0.65-1.15) | 0.91 (0.77-1.09) | 0.92 (0.77-1.10) |  |
| Q3 | 0.99 (0.94-1.04) | 0.99 (0.94-1.04) | 0.99 (0.89-1.11) | 1.00 (0.89-1.11) | 1.04 (0.81-1.34) | 1.05 (0.81-1.36) | 0.85 (0.73-1.00) | 0.86 (0.73-1.01) |  |
| Q4 | 0.98 (0.93-1.04) | 0.98 (0.93-1.04) | 0.96 (0.87-1.06) | 0.97 (0.88-1.07) | 0.91 (0.71-1.18) | 0.92 (0.71-1.19) | 1.02 (0.88-1.18) | 1.01 (0.88-1.17) |  |
| Q5 (highest inequality) | **0.84 (0.80-0.89)** | **0.84 (0.80-0.89)** | **1.13 (1.02-1.25)** | **1.14 (1.03-1.26)** | 0.95 (0.74-1.23) | 0.95 (0.73-1.23) | 0.95 (0.83-1.10) | 0.96 (0.83-1.11) |  |
| Percent unemployed (*P*-interaction^†^ with race/ethnicity = 0.003 for overall and 0.40 for cancer-specific survival) | | | | | | | | |  |
| Q1 (lowest %) | 1.00 (ref) | 1.00 (ref) | 1.00 (ref) | 1.00 (ref) | 1.00 (ref) | 1.00 (ref) | 1.00 (ref) | 1.00 (ref) |  |
| Q2 | 0.98 (0.94-1.03) | 0.98 (0.93-1.03) | 0.99 (0.85-1.15) | 0.98 (0.84-1.15) | 1.06 (0.83-1.35) | 1.06 (0.83-1.37) | 1.03 (0.86-1.24) | 1.02 (0.85-1.23) |  |
| Q3 | **1.06 (1.01-1.11)** | **1.06 (1.01-1.11)** | 1.05 (0.90-1.21) | 1.04 (0.89-1.20) | 1.22 (0.96-1.56) | 1.21 (0.95-1.56) | 0.95 (0.80-1.13) | 0.95 (0.80-1.13) |  |
| Q4 | **1.09 (1.03-1.14)** | **1.09 (1.03-1.14)** | 1.00 (0.87-1.15) | 0.99 (0.86-1.14) | **1.35 (1.09-1.67)** | **1.39 (1.12-1.72)** | 1.08 (0.92-1.26) | 1.07 (0.92-1.26) |  |
| Q5 (highest %) | **1.17 (1.10-1.24)** | **1.17 (1.10-1.24)** | 1.06 (0.92-1.21) | 1.05 (0.92-1.20) | 1.14 (0.92-1.42) | 1.14 (0.92-1.43) | 1.05 (0.91-1.21) | 1.05 (0.90-1.21) |  |
| Percent uninsured (*P*-interaction^†^ with race/ethnicity = 0.52 for overall and 0.98 for cancer-specific survival) | | | | | | | | |  |
| Q1 (lowest %) | 1.00 (ref) | 1.00 (ref) | 1.00 (ref) | 1.00 (ref) | 1.00 (ref) | 1.00 (ref) | 1.00 (ref) | 1.00 (ref) |  |
| Q2 | **1.11 (1.05-1.16)** | **1.11 (1.05-1.16)** | 1.15 (0.95-1.39) | 1.15 (0.95-1.39) | 1.06 (0.80-1.41) | 1.05 (0.78-1.40) | 1.05 (0.84-1.32) | 1.06 (0.85-1.33) |  |
| Q3 | **1.14 (1.08-1.19)** | **1.14 (1.08-1.19)** | 1.07 (0.90-1.28) | 1.07 (0.89-1.29) | 1.17 (0.88-1.55) | 1.18 (0.88-1.58) | 1.07 (0.87-1.32) | 1.10 (0.89-1.35) |  |
| Q4 | **1.17 (1.11-1.23)** | **1.17 (1.11-1.23)** | 1.07 (0.91-1.27) | 1.08 (0.90-1.28) | 1.27 (0.99-1.65) | 1.30 (1.00-1.69) | 1.15 (0.94-1.40) | 1.16 (0.95-1.42) |  |
| Q5 (highest %) | **1.17 (1.10-1.25)** | **1.17 (1.10-1.24)** | 1.04 (0.88-1.24) | 1.04 (0.88-1.24) | **1.34 (1.06-1.70)** | **1.35 (1.06-1.71)** | 1.08 (0.89-1.31) | 1.09 (0.90-1.31) |  |
|  |  |  |  |  |  |  |  |  |  |
| Percent without high school diploma (*P*-interaction^†^ with race/ethnicity = 0.69 for overall and 0.49 for cancer-specific survival) | | | | | | | | |  |
| Q1 (lowest %) | 1.00 (ref) | 1.00 (ref) | 1.00 (ref) | 1.00 (ref) | 1.00 (ref) | 1.00 (ref) | 1.00 (ref) | 1.00 (ref) |  |
| Q2 | **1.13 (1.07-1.18)** | **1.13 (1.08-1.18)** | 1.00 (0.81-1.24) | 1.00 (0.80-1.24) | 1.22 (0.93-1.62) | 1.21 (0.90-1.62) | 0.95 (0.76-1.19) | 0.97 (0.77-1.22) |  |
| Q3 | **1.21 (1.15-1.26)** | **1.21 (1.15-1.27)** | 1.04 (0.85-1.26) | 1.03 (0.84-1.25) | 1.28 (0.96-1.72) | 1.30 (0.97-1.76) | 1.03 (0.82-1.28) | 1.04 (0.83-1.31) |  |
| Q4 | **1.24 (1.18-1.31)** | **1.24 (1.18-1.31)** | 1.04 (0.87-1.26) | 1.04 (0.86-1.26) | **1.36 (1.05-1.74)** | **1.37 (1.06-1.78)** | 1.02 (0.84-1.25) | 1.04 (0.85-1.27) |  |
| Q5 (highest %) | **1.24 (1.16-1.32)** | **1.24 (1.16-1.32)** | 1.09 (0.90-1.32) | 1.09 (0.90-1.32) | **1.55 (1.24-1.94)** | **1.57 (1.25-1.98)** | 1.12 (0.93-1.34) | 1.12 (0.94-1.35) |  |

*Analyses adjusted for age, marital status, primary payer, stage at cancer diagnosis, grade, subtype, year of diagnosis, and treatment status (surgery, chemotherapy, radiation, hormone and immunotherapy). Patients were followed through December 2018.

^†^*P*-value for interaction from likelihood ratio test comparing models with and without interaction between exposure variable and race/ethnicity.

| Supplementary Table 5. Multivariable-adjusted odds ratios (OR) and 95% confidence intervals (CI) for associations of census tract-level socioeconomic variables and breast cancer stage at diagnosis for invasive breast cancer cases diagnosed in 2006-2017 in California^a^ | | | | | | | | | | |
| --- | --- | --- | --- | --- | --- | --- | --- | --- | --- | --- |
| Census tract-level exposure variables | **California – Actual** | | | |  | **California – Synthetic**^c^ | | | |  |
|  | **Stage at diagnosis**^b^ | | | |  | **Stage at diagnosis**^b^ | | | |  |
|  | **Regional** | **Distant** | | **Unknown** |  | **Regional** | | **Distant** | **Unknown** |  |
| Percent below poverty line | | |  | |  |  |  | |  |  |
| Q1 (lowest %) | 1.00 (ref) | 1.00 (ref) | | 1.00 (ref) |  | 1.00 (ref) | | 1.00 (ref) | 1.00 (ref) |  |
| Q2 | 1.02 (0.99-1.05) | **1.09 (1.02-1.17)** | | **1.18 (1.04-1.33)** |  | 1.02 (0.99-1.06) | | **1.12 (1.04-1.20)** | 1.14 (0.99-1.31) |  |
| Q3 | **1.07 (1.04-1.10)** | **1.17 (1.10-1.25)** | | **1.32 (1.17-1.49)** |  | **1.08 (1.04-1.11)** | | **1.23 (1.14-1.32)** | **1.34 (1.13-1.58)** |  |
| Q4 | **1.12 (1.09-1.16)** | **1.25 (1.17-1.33)** | | **1.41 (1.25-1.59)** |  | **1.15 (1.11-1.19)** | | **1.36 (1.27-1.46)** | **1.50 (1.30-1.73)** |  |
| Q5 (highest %) | **1.16 (1.13-1.20)** | **1.39 (1.31-1.48)** | | **1.70 (1.51-1.91)** |  | **1.23 (1.19-1.27)** | | **1.66 (1.55-1.78)** | **1.81 (1.58-2.08)** |  |
| Median household income | | |  | |  |  |  | |  |  |
| Q1 (highest income) | 1.00 (ref) | 1.00 (ref) | | 1.00 (ref) |  | 1.00 (ref) | | 1.00 (ref) | 1.00 (ref) |  |
| Q2 | 1.03 (1.00-1.06) | **1.14 (1.06-1.21)** | | 1.10 (0.97-1.25) |  | 1.04 (1.00-1.07) | | **1.17 (1.08-1.26)** | 1.10 (0.95-1.28) |  |
| Q3 | **1.08 (1.05-1.11)** | **1.15 (1.08-1.23)** | | **1.18 (1.05-1.34)** |  | **1.09 (1.06-1.13)** | | **1.23 (1.14-1.32)** | **1.26 (1.08-1.46)** |  |
| Q4 | **1.14 (1.11-1.18)** | **1.33 (1.24-1.41)** | | **1.42 (1.26-1.60)** |  | **1.16 (1.12-1.19)** | | **1.44 (1.34-1.54)** | **1.55 (1.36-1.77)** |  |
| Q5 (lowest income) | **1.17 (1.13-1.20)** | **1.41 (1.32-1.50)** | | **1.65 (1.47-1.86)** |  | **1.22 (1.18-1.26)** | | **1.69 (1.58-1.81)** | **1.90 (1.66-2.18)** |  |
| Gini index of income inequality | | |  | |  |  |  | |  |  |
| Q1 (lowest inequality) | 1.00 (ref) | 1.00 (ref) | | 1.00 (ref) |  | 1.00 (ref) | | 1.00 (ref) | 1.00 (ref) |  |
| Q2 | 1.02 (0.99-1.05) | 1.05 (0.99-1.12) | | 1.11 (0.99-1.24) |  | 1.02 (0.99-1.05) | | 1.05 (0.99-1.12) | **1.15 (1.01-1.32)** |  |
| Q3 | 1.02 (0.99-1.05) | 1.05 (0.98-1.11) | | 1.08 (0.96-1.21) |  | 1.01 (0.98-1.04) | | 1.06 (0.98-1.13) | 1.12 (0.99-1.26) |  |
| Q4 | 1.00 (0.97-1.03) | 1.05 (0.99-1.11) | | 1.08 (0.97-1.21) |  | 0.98 (0.95-1.01) | | **1.08 (1.01-1.15)** | **1.19 (1.05-1.36)** |  |
| Q5 (highest inequality) | 0.97 (0.94-1.00) | 0.95 (0.89-1.01) | | **1.13 (1.01-1.27)** |  | 0.95 (0.92-0.98) | | 0.97 (0.91-1.04) | **1.22 (1.07-1.39)** |  |
| Percent unemployed | | |  | |  |  |  | |  |  |
| Q1 (lowest %) | 1.00 (ref) | 1.00 (ref) | | 1.00 (ref) |  | 1.00 (ref) | | 1.00 (ref) | 1.00 (ref) |  |
| Q2 | 1.02 (0.99-1.05) | **1.09 (1.02-1.16)** | | **1.17 (1.03-1.32)** |  | 1.03 (1.00-1.07) | | **1.11 (1.03-1.19)** | 1.11 (0.95-1.28) |  |
| Q3 | **1.08 (1.05-1.11)** | **1.13 (1.06-1.20)** | | **1.16 (1.02-1.31)** |  | **1.08 (1.05-1.12)** | | **1.15 (1.07-1.24)** | **1.16 (1.01-1.34)** |  |
| Q4 | **1.12 (1.09-1.16)** | **1.21 (1.13-1.28)** | | **1.33 (1.18-1.50)** |  | **1.14 (1.10-1.18)** | | **1.28 (1.19-1.38)** | **1.30 (1.11-1.52)** |  |
| Q5 (highest %) | **1.14 (1.11-1.17)** | **1.28 (1.20-1.36)** | | **1.52 (1.35-1.70)** |  | **1.17 (1.13-1.22)** | | **1.42 (1.33-1.52)** | **1.53 (1.33-1.76)** |  |
| Percent uninsured | | |  | |  |  |  | |  |  |
| Q1 (lowest %) | 1.00 (ref) | 1.00 (ref) | | 1.00 (ref) |  | 1.00 (ref) | | 1.00 (ref) | 1.00 (ref) |  |
| Q2 | 1.03 (1.00-1.06) | **1.12 (1.05-1.20)** | | **1.33 (1.17-1.51)** |  | **1.05 (1.02-1.09)** | | **1.16 (1.08-1.25)** | **1.26 (1.10-1.45)** |  |
| Q3 | **1.08 (1.05-1.11)** | **1.23 (1.16-1.32)** | | **1.34 (1.19-1.52)** |  | **1.11 (1.08-1.14)** | | **1.31 (1.22-1.40)** | **1.34 (1.17-1.53)** |  |
| Q4 | **1.13 (1.10-1.17)** | **1.37 (1.29-1.46)** | | **1.40 (1.24-1.58)** |  | **1.18 (1.14-1.21)** | | **1.51 (1.41-1.62)** | **1.44 (1.26-1.65)** |  |
| Q5 (highest %) | **1.19 (1.15-1.22)** | **1.39 (1.31-1.49)** | | **1.82 (1.61-2.05)** |  | **1.27 (1.23-1.32)** | | **1.67 (1.56-1.79)** | **1.84 (1.61-2.12)** |  |
| Percent without high school diploma | | |  | |  |  |  | |  |  |
| Q1 (lowest %) | 1.00 (ref) | 1.00 (ref) | | 1.00 (ref) |  | 1.00 (ref) | | 1.00 (ref) | 1.00 (ref) |  |
| Q2 | **1.06 (1.02-1.09)** | **1.14 (1.07-1.22)** | | **1.21 (1.07-1.37)** |  | **1.07 (1.03-1.10)** | | **1.16 (1.07-1.25)** | **1.24 (1.09-1.42)** |  |
| Q3 | **1.11 (1.07-1.14)** | **1.27 (1.20-1.36)** | | **1.22 (1.08-1.38)** |  | **1.12 (1.08-1.15)** | | **1.33 (1.24-1.42)** | **1.25 (1.09-1.43)** |  |
| Q4 | **1.14 (1.11-1.18)** | **1.32 (1.24-1.40)** | | **1.41 (1.25-1.59)** |  | **1.17 (1.14-1.21)** | | **1.44 (1.34-1.54)** | **1.45 (1.26-1.65)** |  |
| Q5 (highest %) | **1.20 (1.16-1.24)** | **1.47 (1.38-1.57)** | | **1.65 (1.46-1.86)** |  | **1.27 (1.23-1.31)** | | **1.71 (1.59-1.84)** | **1.74 (1.50-2.02)** |  |

^a^Analyses adjusted for age, race, ethnicity, marital status, primary payer, and year of diagnosis.

^b^Reference category for stage at diagnosis is local stage disease.

^c^Results from 10 iterations pooled using SAS MIANALYZE procedure.

| Supplementary Table 6. Multivariable-adjusted odds ratios (OR) and 95% confidence intervals (CI) for associations of census tract-level socioeconomic variables and breast tumor differentiation for invasive breast cancer cases diagnosed in 2006-2017 in California^a^ | | | | | | | | | | |
| --- | --- | --- | --- | --- | --- | --- | --- | --- | --- | --- |
| Census tract-level exposure variables | **California – Actual** | |  | | | **California – Synthetic**^c^ | | | | |
|  | **Tumor differentiation**^b^ | |  | | | **Tumor differentiation**^b^ | | | | |
|  | **Poorly differentiated/ undifferentiated** | **Unknown** | | |  | | **Poorly differentiated/ undifferentiated** | | | **Unknown** |
| Percent below poverty line | |  |  | | |  | | |  |  |
| Q1 (lowest %) | 1.00 (ref) | 1.00 (ref) | | |  | | | 1.00 (ref) | | 1.00 (ref) |
| Q2 | **1.04 (1.01-1.07)** | 1.05 (0.99-1.12) | | |  | | | 1.03 (0.99-1.07) | | 1.04 (0.97-1.11) |
| Q3 | **1.09 (1.06-1.13)** | **1.17 (1.10-1.25)** | | |  | | | **1.06 (1.02-1.10)** | | **1.11 (1.03-1.21)** |
| Q4 | **1.19 (1.15-1.23)** | **1.34 (1.26-1.42)** | | |  | | | **1.09 (1.04-1.13)** | | **1.19 (1.09-1.30)** |
| Q5 (highest %) | **1.29 (1.25-1.33)** | **1.50 (1.41-1.59)** | | |  | | | **1.15 (1.11-1.19)** | | **1.31 (1.21-1.42)** |
| Median household income | |  |  | | |  | | |  |  |
| Q1 (highest income) | 1.00 (ref) | 1.00 (ref) | | |  | | | 1.00 (ref) | | 1.00 (ref) |
| Q2 | **1.09 (1.06-1.13)** | **1.12 (1.05-1.19)** | | |  | | | **1.05 (1.01-1.09)** | | 1.06 (0.99-1.14) |
| Q3 | **1.16 (1.12-1.20)** | **1.21 (1.14-1.29)** | | |  | | | **1.08 (1.04-1.12)** | | **1.14 (1.05-1.23)** |
| Q4 | **1.26 (1.22-1.30)** | **1.41 (1.33-1.50)** | | |  | | | **1.13 (1.09-1.17)** | | **1.28 (1.19-1.39)** |
| Q5 (lowest income) | **1.36 (1.31-1.40)** | **1.55 (1.46-1.65)** | | |  | | | **1.18 (1.13-1.22)** | | **1.36 (1.27-1.45)** |
| Gini index of income inequality | |  |  | | |  | | |  |  |
| Q1 (lowest inequality) | 1.00 (ref) | 1.00 (ref) | | |  | | | 1.00 (ref) | | 1.00 (ref) |
| Q2 | 1.02 (0.99-1.06) | 1.04 (0.98-1.10) | | |  | | | 1.00 (0.96-1.04) | | 1.02 (0.92-1.13) |
| Q3 | 1.02 (0.99-1.05) | 1.03 (0.97-1.09) | | |  | | | 1.00 (0.96-1.04) | | 0.99 (0.92-1.07) |
| Q4 | 1.03 (0.99-1.06) | 1.05 (0.99-1.12) | | |  | | | 1.00 (0.95-1.06) | | 1.01 (0.94-1.09) |
| Q5 (highest inequality) | 0.97 (0.94-1.00) | 0.94 (0.88-1.00) | | |  | | | 0.97 (0.93-1.02) | | 0.96 (0.89-1.04) |
| Percent unemployed | |  |  | | |  | | |  |  |
| Q1 (lowest %) | 1.00 (ref) | 1.00 (ref) | | |  | | | 1.00 (ref) | | 1.00 (ref) |
| Q2 | 1.03 (1.00-1.07) | 1.06 (1.00-1.12) | | |  | | | 1.03 (0.99-1.06) | | 1.05 (0.98-1.13) |
| Q3 | **1.09 (1.05-1.12)** | **1.15 (1.08-1.22)** | | |  | | | **1.06 (1.02-1.10)** | | **1.11 (1.04-1.19)** |
| Q4 | **1.15 (1.12-1.19)** | **1.27 (1.19-1.34)** | | |  | | | **1.09 (1.05-1.14)** | | **1.21 (1.12-1.30)** |
| Q5 (highest %) | **1.20 (1.17-1.24)** | **1.50 (1.41-1.59)** | | |  | | | **1.14 (1.10-1.19)** | | **1.39 (1.30-1.48)** |
| Percent uninsured | |  |  | | |  | | |  |  |
| Q1 (lowest %) | 1.00 (ref) | 1.00 (ref) | |  | | | 1.00 (ref) | | | 1.00 (ref) |
| Q2 | **1.12 (1.08-1.15)** | **1.17 (1.10-1.24)** | |  | | | **1.07 (1.02-1.11)** | | | **1.12 (1.04-1.20)** |
| Q3 | **1.17 (1.14-1.21)** | **1.30 (1.23-1.39)** | |  | | | **1.10 (1.05-1.15)** | | | **1.19 (1.10-1.28)** |
| Q4 | **1.25 (1.21-1.29)** | **1.39 (1.31-1.48)** | |  | | | **1.15 (1.11-1.20)** | | | **1.25 (1.16-1.35)** |
| Q5 (highest %) | **1.39 (1.35-1.44)** | **1.49 (1.40-1.59)** | |  | | | **1.21 (1.16-1.27)** | | | **1.30 (1.20-1.40)** |
|  | |  |  | | |  | | |  |  |
| Percent without high school diploma | |  |  | | |  | | |  |  |
| Q1 (lowest %) | 1.00 (ref) | 1.00 (ref) | |  | | | 1.00 (ref) | | | 1.00 (ref) |
| Q2 | **1.08 (1.04-1.11)** | **1.18 (1.11-1.25)** | |  | | | 1.03 (0.99-1.07) | | | **1.11 (1.02-1.20)** |
| Q3 | **1.15 (1.11-1.19)** | **1.31 (1.23-1.39)** | |  | | | **1.08 (1.03-1.13)** | | | **1.20 (1.10-1.31)** |
| Q4 | **1.24 (1.20-1.28)** | **1.42 (1.34-1.51)** | |  | | | **1.13 (1.09-1.18)** | | | **1.27 (1.18-1.38)** |
| Q5 (highest %) | **1.37 (1.32-1.41)** | **1.56 (1.46-1.66)** | |  | | | **1.17 (1.13-1.23)** | | | **1.32 (1.21-1.45)** |

^a^Analyses adjusted for age, race, ethnicity, and year of diagnosis.

^b^Reference category for tumor differentiation is well/moderately well differentiated.

^c^Results from 10 iterations pooled using SAS MIANALYZE procedure.

| Supplementary Table 7. Multivariable-adjusted odds ratios (OR) and 95% confidence intervals (CI) for associations of census tract-level socioeconomic variables and breast tumor subtype for invasive breast cancer cases diagnosed in 2010-2017 in California^a^ | | | | | | | | | | | | | | |  |
| --- | --- | --- | --- | --- | --- | --- | --- | --- | --- | --- | --- | --- | --- | --- | --- |
| Census tract-level exposure variables | **California – Actual** | | | | |  | | | **California – Synthetic**^c^ | | | | | | |
|  | **Tumor subtype**^b^ | | | | |  | | | **Tumor subtype**^b^ | | | | | | |
|  | **HR+/HER2+**  **(Luminal B)** | **HR-/HER2+**  **(HER2-enriched)** | | **HR-/HER2-**  **(triple negative)** | | |  | | | **HR+/HER2+**  **(Luminal B)** | | | **HR-/HER2+**  **(HER2-enriched)** | **HR-/HER2-**  **(triple negative)** | |
| Percent below poverty line | | |  | |  | | |  | | | |  | |  | |
| Q1 (lowest %) | 1.00 (ref) | 1.00 (ref) | | 1.00 (ref) | | |  | | | | 1.00 (ref) | | 1.00 (ref) | 1.00 (ref) | |
| Q2 | 0.99 (0.94-1.05) | 1.06 (0.98-1.15) | | 1.05 (0.99-1.11) | | |  | | | | 1.02 (0.94-1.10) | | 1.02 (0.90-1.16) | 1.03 (0.96-1.10) | |
| Q3 | 1.05 (0.99-1.10) | **1.12 (1.03-1.21)** | | **1.13 (1.07-1.19)** | | |  | | | | 1.05 (0.98-1.12) | | 1.05 (0.95-1.17) | 1.06 (0.99-1.13) | |
| Q4 | **1.07 (1.01-1.13)** | **1.20 (1.11-1.30)** | | **1.16 (1.10-1.23)** | | |  | | | | 1.06 (1.00-1.12) | | 1.08 (0.97-1.20) | 1.08 (0.99-1.18) | |
| Q5 (highest %) | **1.13 (1.07-1.19)** | **1.33 (1.23-1.44)** | | **1.28 (1.21-1.36)** | | |  | | | | 1.08 (1.00-1.16) | | **1.17 (1.05-1.31)** | **1.14 (1.07-1.22)** | |
| Median household income | | |  | |  | | |  | | | |  | |  | |
| Q1 (highest income) | 1.00 (ref) | 1.00 (ref) | | 1.00 (ref) | | |  | | | | 1.00 (ref) | | 1.00 (ref) | 1.00 (ref) | |
| Q2 | 1.04 (0.99-1.10) | **1.09 (1.01-1.18)** | | **1.07 (1.01-1.13)** | | |  | | | | 1.03 (0.96-1.11) | | 1.02 (0.92-1.13) | 1.04 (0.97-1.12) | |
| Q3 | **1.08 (1.02-1.14)** | **1.18 (1.09-1.27)** | | **1.12 (1.06-1.19)** | | |  | | | | 1.04 (0.96-1.11) | | 1.04 (0.94-1.15) | 1.07 (0.99-1.17) | |
| Q4 | **1.11 (1.05-1.17)** | **1.22 (1.13-1.32)** | | **1.23 (1.16-1.30)** | | |  | | | | **1.07 (1.01-1.13)** | | **1.12 (1.01-1.24)** | **1.13 (1.05-1.22)** | |
| Q5 (lowest income) | **1.17 (1.11-1.24)** | **1.36 (1.25-1.47)** | | **1.29 (1.22-1.36)** | | |  | | | | **1.08 (1.02-1.15)** | | **1.16 (1.05-1.29)** | **1.16 (1.07-1.25)** | |
| Gini index of income inequality | | |  | |  | | |  | | | |  | |  | |
| Q1 (lowest inequality) | 1.00 (ref) | 1.00 (ref) | | 1.00 (ref) | | |  | | | | 1.00 (ref) | | 1.00 (ref) | 1.00 (ref) | |
| Q2 | 1.03 (0.98-1.09) | 1.05 (0.97-1.13) | | 1.01 (0.96-1.07) | | |  | | | | 0.99 (0.92-1.07) | | 0.98 (0.86-1.12) | 1.01 (0.95-1.08) | |
| Q3 | 0.97 (0.92-1.02) | 1.07 (1.00-1.15) | | 0.98 (0.93-1.03) | | |  | | | | 0.99 (0.93-1.06) | | 0.99 (0.87-1.13) | 1.00 (0.93-1.09) | |
| Q4 | 1.00 (0.95-1.05) | 0.99 (0.92-1.07) | | 0.98 (0.93-1.04) | | |  | | | | 0.99 (0.92-1.07) | | 0.98 (0.87-1.11) | 0.99 (0.92-1.08) | |
| Q5 (highest inequality) | 1.01 (0.96-1.06) | 0.95 (0.88-1.02) | | **0.90 (0.86-0.95)** | | |  | | | | 0.99 (0.93-1.05) | | 0.98 (0.89-1.08) | 0.96 (0.89-1.03) | |
| Percent unemployed | | |  | |  | | |  | | | |  | |  | |
| Q1 (lowest %) | 1.00 (ref) | 1.00 (ref) | | 1.00 (ref) | | |  | | | | 1.00 (ref) | | 1.00 (ref) | 1.00 (ref) | |
| Q2 | 1.00 (0.95-1.05) | 1.06 (0.98-1.14) | | **1.12 (1.06-1.18)** | | |  | | | | 1.02 (0.94-1.09) | | 1.03 (0.93-1.13) | 1.00 (0.93-1.08) | |
| Q3 | **1.08 (1.02-1.14)** | **1.09 (1.01-1.18)** | | **1.13 (1.07-1.19)** | | |  | | | | 1.03 (0.97-1.11) | | 1.03 (0.92-1.16) | 1.03 (0.94-1.13) | |
| Q4 | **1.11 (1.05-1.17)** | **1.16 (1.07-1.25)** | | **1.19 (1.13-1.26)** | | |  | | | | 1.05 (0.96-1.15) | | 1.07 (0.93-1.23) | 1.07 (0.99-1.16) | |
| Q5 (highest %) | **1.06 (1.01-1.12)** | **1.26 (1.16-1.36)** | | **1.26 (1.19-1.33)** | | |  | | | | 1.06 (0.96-1.16) | | **1.16 (1.03-1.31)** | **1.13 (1.06-1.21)** | |
| Percent uninsured | | |  | |  | | |  | | | |  | |  | |
| Q1 (lowest %) | 1.00 (ref) | 1.00 (ref) | | 1.00 (ref) | | |  | | | | 1.00 (ref) | | 1.00 (ref) | 1.00 (ref) | |
| Q2 | 1.02 (0.96-1.07) | **1.12 (1.03-1.21)** | | **1.13 (1.07-1.19)** | | |  | | | | 1.06 (0.99-1.13) | | 1.05 (0.93-1.18) | 1.05 (0.96-1.15) | |
| Q3 | **1.10 (1.04-1.16)** | **1.19 (1.10-1.29)** | | **1.18 (1.12-1.25)** | | |  | | | | **1.07 (1.01-1.14)** | | 1.08 (0.95-1.22) | 1.09 (1.00-1.18) | |
| Q4 | **1.10 (1.04-1.16)** | **1.29 (1.19-1.39)** | | **1.22 (1.15-1.29)** | | |  | | | | **1.09 (1.01-1.17)** | | 1.10 (0.98-1.22) | **1.12 (1.02-1.23)** | |
| Q5 (highest %) | **1.18 (1.12-1.25)** | **1.34 (1.23-1.45)** | | **1.28 (1.21-1.36)** | | |  | | | | **1.11 (1.03-1.19)** | | **1.16 (1.03-1.31)** | **1.13 (1.03-1.25)** | |
| Percent without high school diploma | | |  | |  | | |  | | | |  | |  | |
| Q1 (lowest %) | 1.00 (ref) | 1.00 (ref) | | 1.00 (ref) | | |  | | | | 1.00 (ref) | | 1.00 (ref) | 1.00 (ref) | |
| Q2 | 1.05 (0.99-1.10) | **1.10 (1.02-1.19)** | | **1.10 (1.04-1.16)** | | |  | | | | 1.03 (0.95-1.11) | | 1.03 (0.91-1.17) | 1.04 (0.97-1.13) | |
| Q3 | **1.09 (1.04-1.15)** | **1.22 (1.13-1.32)** | | **1.18 (1.11-1.25)** | | |  | | | | 1.05 (0.97-1.14) | | 1.07 (0.96-1.19) | 1.09 (1.00-1.19) | |
| Q4 | **1.12 (1.06-1.19)** | **1.24 (1.15-1.35)** | | **1.25 (1.18-1.32)** | | |  | | | | 1.07 (0.99-1.16) | | 1.09 (0.99-1.20) | **1.13 (1.05-1.22)** | |
| Q5 (highest %) | **1.18 (1.12-1.25)** | **1.38 (1.27-1.49)** | | **1.37 (1.29-1.45)** | | |  | | | | 1.08 (0.99-1.16) | | **1.16 (1.04-1.30)** | **1.16 (1.06-1.27)** | |

^a^Analyses adjusted for age, race, ethnicity, and year of diagnosis.

^b^Reference category for tumor subtype is HR+/HER2- (Luminal A). Cases diagnosed before 2010 were excluded and cases with missing or unknown HR or HER2 status are omitted from table.

^c^Results from 10 iterations pooled using SAS MIANALYZE procedure.

| Supplementary Table 8. Multivariable-adjusted hazard ratios (HR) and 95% confidence intervals (CI) for associations of census tract-level socioeconomic variables and overall and cancer-specific survival for invasive breast cancer cases diagnosed in 2006-2016 in California^a^ | | | | | | | | | | | | | |  |
| --- | --- | --- | --- | --- | --- | --- | --- | --- | --- | --- | --- | --- | --- | --- |
| Census tract-level exposure variables | **California – Actual** | | | |  | | | **California – Synthetic**^b^ | | | | | | |
|  | **Overall survival** | **Cancer-specific survival** | | | |  | | | **Overall survival** | | | **Cancer-specific survival** | | |
| Percent below poverty line | | |  |  | | |  | | | |  |  |  |  |
| Q1 (lowest %) | 1.00 (ref) | 1.00 (ref) | | | |  | | | | 1.00 (ref) | | 1.00 (ref) | | |
| Q2 | **1.06 (1.02-1.10)** | **1.06 (1.01-1.12)** | | | |  | | | | 1.00 (0.95-1.06) | | 1.00 (0.94-1.07) | | |
| Q3 | **1.11 (1.06-1.15)** | **1.10 (1.04-1.15)** | | | |  | | | | 1.03 (0.98-1.08) | | 1.03 (0.96-1.10) | | |
| Q4 | **1.21 (1.16-1.25)** | **1.20 (1.14-1.26)** | | | |  | | | | **1.06 (1.01-1.11)** | | 1.06 (0.99-1.14) | | |
| Q5 (highest %) | **1.31 (1.26-1.36)** | **1.30 (1.24-1.36)** | | | |  | | | | **1.11 (1.06-1.16)** | | **1.11 (1.05-1.17)** | | |
| Median household income | | |  |  | | |  | | | |  |  |  |  |
| Q1 (highest income) | 1.00 (ref) | 1.00 (ref) | | | |  | | | | 1.00 (ref) | | 1.00 (ref) | | |
| Q2 | **1.12 (1.08-1.17)** | **1.10 (1.05-1.16)** | | | |  | | | | 1.02 (0.96-1.08) | | 1.01 (0.94-1.09) | | |
| Q3 | **1.20 (1.16-1.25)** | **1.18 (1.12-1.24)** | | | |  | | | | 1.05 (1.00-1.11) | | 1.05 (0.99-1.12) | | |
| Q4 | **1.30 (1.25-1.35)** | **1.27 (1.21-1.33)** | | | |  | | | | **1.09 (1.03-1.14)** | | **1.09 (1.02-1.16)** | | |
| Q5 (lowest income) | **1.38 (1.33-1.44)** | **1.35 (1.29-1.42)** | | | |  | | | | **1.14 (1.09-1.20)** | | **1.14 (1.07-1.21)** | | |
| Gini index of income inequality | | |  |  | | |  | | | |  |  |  |  |
| Q1 (lowest inequality) | 1.00 (ref) | 1.00 (ref) | | | |  | | | | 1.00 (ref) | | 1.00 (ref) | | |
| Q2 | 1.01 (0.98-1.05) | 0.98 (0.93-1.02) | | | |  | | | | 0.99 (0.95-1.03) | | 0.99 (0.93-1.06) | | |
| Q3 | 1.01 (0.98-1.05) | 0.99 (0.94-1.04) | | | |  | | | | 0.98 (0.93-1.03) | | 0.98 (0.93-1.04) | | |
| Q4 | 0.99 (0.96-1.03) | 0.98 (0.94-1.03) | | | |  | | | | 0.98 (0.94-1.03) | | 0.99 (0.93-1.06) | | |
| Q5 (highest inequality) | **0.92 (0.89-0.96)** | **0.91 (0.87-0.96)** | | | |  | | | | 0.96 (0.92-1.00) | | 0.97 (0.91-1.03) | | |
| Percent unemployed | | |  |  | | |  | | | |  |  |  |  |
| Q1 (lowest %) | 1.00 (ref) | 1.00 (ref) | | | |  | | | | 1.00 (ref) | | 1.00 (ref) | | |
| Q2 | **1.08 (1.04-1.13)** | **1.09 (1.04-1.14)** | | | |  | | | | 1.03 (0.97-1.08) | | 1.03 (0.96-1.10) | | |
| Q3 | **1.12 (1.08-1.17)** | **1.13 (1.07-1.19)** | | | |  | | | | 1.04 (0.99-1.10) | | 1.05 (0.99-1.12) | | |
| Q4 | **1.21 (1.16-1.25)** | **1.22 (1.16-1.28)** | | | |  | | | | **1.09 (1.04-1.14)** | | **1.09 (1.03-1.16)** | | |
| Q5 (highest %) | **1.31 (1.26-1.36)** | **1.29 (1.23-1.35)** | | | |  | | | | **1.13 (1.06-1.20)** | | **1.12 (1.04-1.20)** | | |
| Percent uninsured | | |  |  | | |  | | | |  |  |  |  |
| Q1 (lowest %) | 1.00 (ref) | 1.00 (ref) | | | |  | | | | 1.00 (ref) | | 1.00 (ref) | | |
| Q2 | **1.13 (1.09-1.18)** | **1.10 (1.05-1.16)** | | | |  | | | | 1.02 (0.97-1.07) | | 1.02 (0.94-1.10) | | |
| Q3 | **1.23 (1.19-1.28)** | **1.23 (1.17-1.29)** | | | |  | | | | **1.07 (1.03-1.12)** | | 1.08 (1.00-1.15) | | |
| Q4 | **1.29 (1.24-1.34)** | **1.27 (1.21-1.34)** | | | |  | | | | **1.09 (1.04-1.15)** | | **1.10 (1.02-1.18)** | | |
| Q5 (highest %) | **1.36 (1.31-1.41)** | **1.37 (1.30-1.43)** | | | |  | | | | **1.09 (1.04-1.14)** | | **1.11 (1.03-1.19)** | |  |
| Percent without high school diploma | | |  |  | | |  | | | |  |  |  |  |
| Q1 (lowest %) | 1.00 (ref) | 1.00 (ref) | | | |  | | | | 1.00 (ref) | | 1.00 (ref) | | |
| Q2 | **1.12 (1.08-1.16)** | **1.10 (1.05-1.16)** | | | |  | | | | 1.04 (0.98-1.10) | | 1.03 (0.96-1.12) | | |
| Q3 | **1.23 (1.19-1.28)** | **1.23 (1.17-1.29)** | | | |  | | | | **1.08 (1.03-1.14)** | | **1.08 (1.02-1.15)** | | |
| Q4 | **1.36 (1.31-1.41)** | **1.36 (1.30-1.43)** | | | |  | | | | **1.10 (1.05-1.15)** | | **1.10 (1.04-1.17)** | | |
| Q5 (highest %) | **1.41 (1.35-1.47)** | **1.38 (1.31-1.46)** | | | |  | | | | **1.12 (1.05-1.18)** | | **1.12 (1.05-1.20)** | | |

^a^Analyses adjusted for age, race, ethnicity, marital status, primary payer, stage at cancer diagnosis, grade, subtype, year of diagnosis, and treatment status (surgery, chemotherapy, radiation). Patients were followed through December 2017.

^b^Results from 10 iterations pooled using SAS MIANALYZE procedure.
